# Metabolomic analysis on the mechanism of nanoselenium alleviating cadmium stress and improving the pepper nutritional value

**DOI:** 10.1186/s12951-022-01739-5

**Published:** 2022-12-10

**Authors:** Dong Li, Chunran Zhou, Jia-Qi Li, Qinyong Dong, Peijuan Miao, Yongxi Lin, Haiyan Cheng, Yuwei Wang, Luna Luo, Canping Pan

**Affiliations:** 1 Innovation Center of Pesticide Research, Department of Applied Chemistry, College of Science, China Agricultural University, Beijing, 100193 China; 2 Key Laboratory of Green Prevention and Control of Tropical Plant Diseases and Pests, College of Plant Protection, Ministry of Education, Hainan University, Haikou, Hainan 570228 People’s Republic of China; 3 Key Laboratory of Tropical Fruits and Vegetables Quality and Safety for State Market Regulation, Haikou, 570311 China

**Keywords:** Cd, Nano-selenium, Nutrient quality, Pepper plants, Stress-resistant pathway

## Abstract

Selenium (Se) maintains soil-plant homeostasis in the rhizosphere and regulates signaling molecules to mitigate cadmium (Cd) toxicity. However, there has been no systematic investigation of the effects of nano-selenium (nano-Se) on the regulation of non-target metabolites and nutritional components in pepper plants under Cd stress. This study investigated the effects of Cd-contaminated soil stress and nano-Se (1, 5, and 20 mg/L) on the metabolic mechanism, fruit nutritional quality, and volatile organic compounds (VOCs) composition of pepper plants. The screening of differential metabolites in roots and fruit showed that most were involved in amino acid metabolism and capsaicin production. Amino acids in roots (Pro, Trp, Arg, and Gln) and fruits (Phe, Glu, Pro, Arg, Trp, and Gln) were dramatically elevated by nano-Se biofortification. The expression of genes of the phenylpropane-branched fatty acid pathway (*BCAT*, *Fat*, *AT3*, *HCT*, and *Kas*) was induced by nano-Se (5 mg/L), increasing the levels of capsaicin (29.6%), nordihydrocapsaicin (44.2%), and dihydrocapsaicin (45.3%). VOCs (amyl alcohol, linalool oxide, E-2-heptaldehyde, 2-hexenal, ethyl crotonate, and 2-butanone) related to crop resistance and quality were markedly increased in correspondence with the nano-Se concentration. Therefore, nano-Se can improve the health of pepper plants by regulating the capsaicin metabolic pathway and modulating both amino acid and VOC contents.

**Graphical Abstract:**

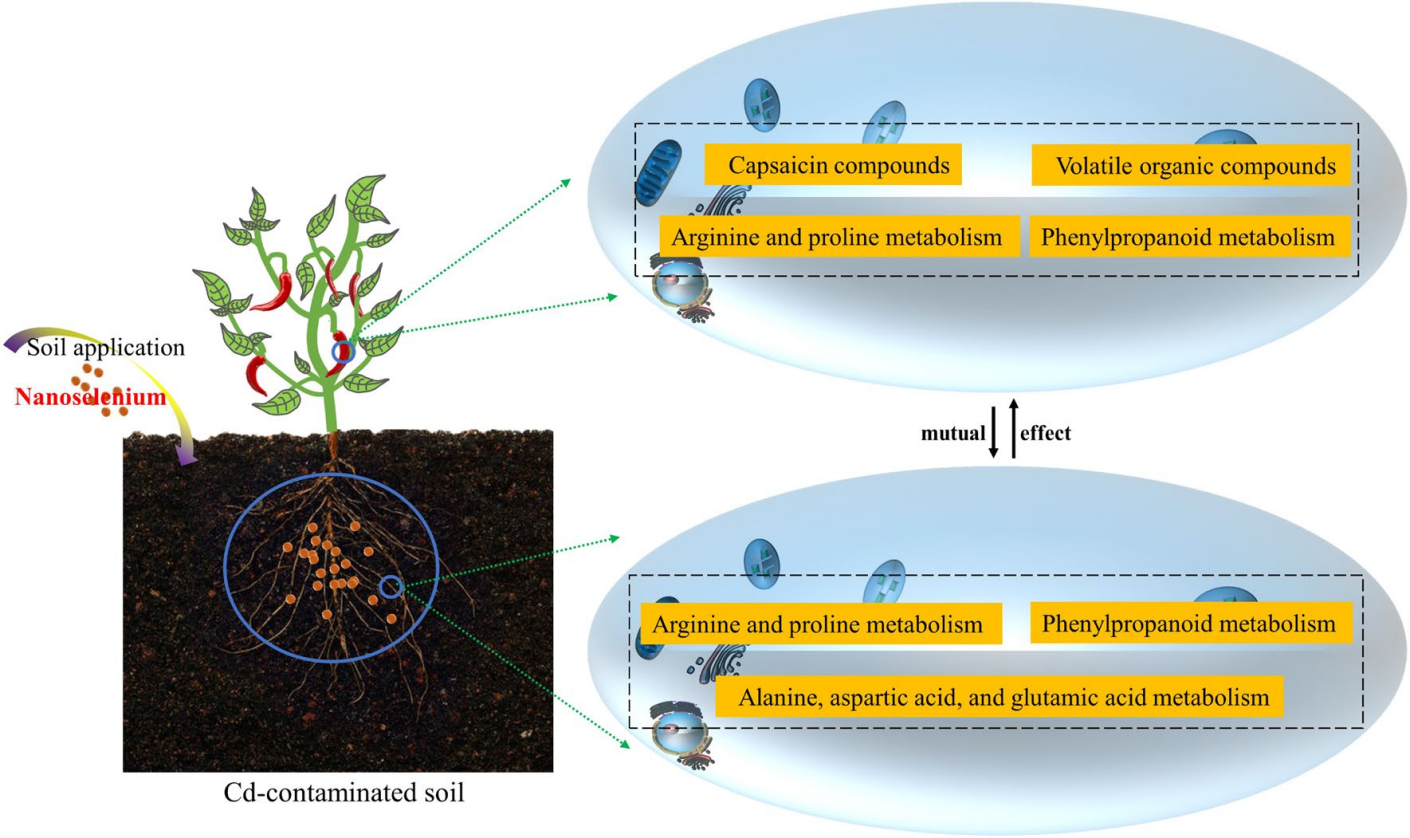

**Supplementary Information:**

The online version contains supplementary material available at 10.1186/s12951-022-01739-5.

## Introduction

Cadmium (Cd) is a non-essential trace element prevalent in plants. Human activities such as the disposal of municipal waste, smelting, mining, metal manufacturing, and the application of synthetic phosphate fertilizer have raised the concentrations of Cd in the environment, with health-related consequences due to Cd carcinogenicity [1]. Crop plants are susceptible to Cd toxicity, which limits nutrient and water absorption and translocation, increases oxidative damage, affects plant metabolism, and impairs plant morphology and physiology [2]. Cd stress causes the over-production of reactive oxygen species (ROS) in the plant, potentially leading to protein oxidation, enzyme inhibition, DNA and RNA damage, and MDA generation from membrane lipid peroxidation [3]. It also lowers chlorophyll production, photosynthesis, and respiration, thus reducing both plant output and quality [4]. As a result, Cd toxicity can also affect soil-microbial-plant interactions, seed germination, growth and development, photosynthesis, and nutrient production in crops [5–7].

Few studies have addressed the issue of Cd toxicity in ensuring crop safety and quality, increasing both nutritional levels and agricultural production. Plants adapt to Cd stress by storing and accumulating Cd through binding to amino acids, peptides, and proteins [8]. Plants also produce stress-related signaling molecules and activate stress-related pathways, including both primary (carbohydrates and amino acids) and secondary (plant hormones) metabolic pathways [9]. Signaling compounds produced in response to Cd stress participate in cellular responses to minimize Cd toxicity. Many strategies have been developed to reduce metal accumulation and toxicity in plants, including physical remediation (in situ fixation), chemical remediation (soil amendments), and biological remediation (microbial remediation) [10]. Advances in nanotechnology and their agriculture applications have led to significant innovations with the development of efficient and cost-effective products that can be applied to crops and transform the agricultural industry [11].

The use of Se to minimize metal uptake and toxicity in plants is gaining increasing attention. Nano-selenium (nano-Se) can enhance plant physiological and biochemical processes and improve crop growth, yield, and quality. It can ameliorate both biological stress (pests and diseases) and abiotic stress (salt and heavy metals) and boost the nutritional value of crops [12–14]. As described in a previous publication, we found that nano-Se treatment of Cd-stressed pepper plants enhanced growth and development, improved root ultrastructure, increased the expression of metabolic and lignin pathway-related genes, and reduced Cd accumulation and toxicity [15]. In addition, nano-Se treatment maintained the optimum balance between the plant and soil in the rhizosphere by improving rhizosphere soil quality (environmental index, enzyme activity, microbial communities, metabolites, Se and Cd morphology) and the distribution of soil-plant signaling molecules (phytohormones and phenolic acids). The study investigated links between microbial diversity, target metabolites, and levels of gene expression in the rhizosphere soil and pepper plants [16]. However, there has been no comprehensive investigation into the role of nano-Se in the regulation of non-target metabolites and nutritional components in pepper plants under Cd stress.

In this study, non-target metabolic components, fruit quality, and volatile organic compounds (VOCs) in different parts of pepper plants were evaluated under conditions of Cd stress and nano-Se intervention. The associations between metabolic pathways and the nutritional quality of the plants were explored using broad-target metabolomics and the verification of target metabolites. The principal objective was the elucidation of the internal mechanism by which nano-Se ameliorates crop stress resistance and fruit nutrient quality.

## Materials and methods

### Plant cultivation and experimental conditions

Plants were grown in a greenhouse at the China Agricultural University (Beijing, China) under the following growing conditions: Photon flux (250–300 µmol m^− 2^ s^− 1^); temperature of 25/18 °C (day/night); light/dark cycle of 12/12 h; 75–85% humidity. Cd-contaminated soil was collected from a vegetable field in the city of Changsha, Hunan, China (28°26′ N, 113°03′ E); the soil properties were characterized and described in earlier research [16]. Varying amounts of nano-Se were dissolved in pure water (0, 1, 5, and 20 mg/L) before application to the Cd-contaminated soil. The nano-Se solution was then used to spray the soil until the moisture content reached 10%. The blended soil stabilized after seven days. Pepper seeds (*Capsicum annuum* L. var. *conoides* (Mill.) Irish) were immersed in deionized water containing 0.5% NaClO for 2 h, and the seed was then planted in a plastic container (2 L) containing Cd-contaminated soil. There were four distinct treatments in all, each with eight repetitions. Our previous articles have described nano-Se in detail [17]. The application of nano-Se (1, 5, and 20 mg/L) was found to significantly increase Cd levels in the root while decreasing those in the pepper tissues (stems, leaves, and fruit) relative to the control treatment [16]. Based on these previous findings, the present investigation focused on the mechanisms by which nano-Se reduces Cd stress and improves the nutritional value of the pepper fruit. To ensure that all treatments were constant, regular fertilization was conducted throughout the growing season. The dosage of nano-Se was constant and consistent application was conducted throughout the treatment procedure. After three months of cultivation, the samples were washed with water to remove any Se that may have remained on the surface. Then, pepper fruits, leaves, stems, roots, and rhizosphere soil were collected and immediately sent to the laboratory. To guarantee adequate samples for metabolomics analysis, 20 samples were collected from each treatment with all samples taken under identical conditions. The samples were stored at a temperature of − 80 °C and kept as uniform in size as possible.

### Amino acid analyses

The roots, leaves, and freeze-dried peppers (20 mg) were combined with 1 mL of purified water. The solution was ultrasonicated for 30 min before centrifugation at 10 000 rpm for 10 min. Before testing, the supernatant was derivatized using 6-aminoquinolyl-N-hydroxy-succinimidyl carbamate (AQC). The amino acid contents were determined using liquid chromatography-high resolution mass spectrometry (LC-HRMS) and quantitatively analyzed using the Xcalibur program (Thermo Scientific, MA, USA). Previous research articles have described the specific showed instrument parameters and settings [18].

### Wide-target metabolomics detection of root and pepper fruit

Extraction of metabolites: 50 mg of each sample was accurately weighed and transferred to an Eppendorf tube containing 700 µL of extract solution (methanol: water = 3:1, containing internal standard). The material was vortexed for 30 s, homogenized for 4 min at 35 Hz, and sonicated for 5 min in a cold bath. The mixture was homogenized and sonicated three times. The samples were then agitated on a shaker overnight at 4 ℃ and centrifuged at 4 ℃ for 15 min at 12 000 rpm (RCF = 13,800 (×g), R = 8.6 cm). The supernatant was carefully filtered through a 0.22-µm microporous membrane, and the remaining supernatants were diluted twice with an extract solution (methanol: water = 3:1, V/V, including internal standard) and vortexed for 30 s before being transferred to 2-mL glass vials and pooled as QC samples (40 µL). All solutions were kept at −80 ℃ until the UHPLC-MS analysis.

UHPLC- MS analysis: the UHPLC separation was carried out using an EXIONLC System (Sciex). The mobile phases A and B were water with 0.1% formic acid and acetonitrile, respectively. The column’s temperature was set at 40 ℃. The temperature of the auto-sampler was set at 4 ℃, and the injection volume was 2 µL. The flow rate was set at 0.4 mL/min. The following gradient was used for elution: 98% A, 0 min; 98% A, 0.5 min; 50% A, 10 min; 5% A, 11 min; 5% A, 13 min; 98% A, 13.1 min; 98% A, 15 min. A Sciex QTrap 6500+ (Sciex Technologies) was used for assay development with the settings: IonSpray voltage, + 5500/− 4500 V; curtain gas, 35 psi; temperature, 400^o^C; ion source gas 1, 60 psi; ion source gas 2, 60 psi; DP, ± 100 V.

Data preprocessing and annotation: SCIEX Analyst Work Station Software was used to capture and process the MRM data (Version 1.6.3). MSconventer was applied to convert MS raw data (.wiff) files to the TXT format. Peak identification and labeling were carried out with the use of In-house R software and a database.

### Capsaicin compound determinations

This followed our previous method with suitable updates [19]. Freeze-dried powdered pepper fruit (20 mg) was homogenized in 1 mL extracting solution (ethanol: water = 1:1, V: V). The homogenate was shaken for 5 min before ultrasonication for 60 min and centrifugation at 10 000 g for 5 min at 4 °C. The supernatant was filtered through a 0.22-µm nylon filter before analysis using an Agilent G6465B triple quadrupole UPLC-MS/MS coupled with an HPLC reverse phase C18 column (Eclipse Plus C18 2.1 × 50 mm, 1.8 μm). The flow rate was 0.4 mL/min. The mobile phases A and B were acetonitrile and 0.1% formic acid in the water, respectively. The gradient elution was 5% A for 0 min, 100% A for 4 min, 5% A for 4.1 min, and 5% A for 5.3 min. The MS was performed using multiple reaction monitoring modes (MRM) and positive electrospray ionization. Additional file 1: Table S6 lists the specific instrumental parameters.

### Total RNA extraction and real-time PCR quantification

Total RNA was extracted from pepper fruit (control, nano-Se1, nano-Se5, and nano-Se20) using an RNAprep pure Plant Kit, according to the manufacturer’s instructions (Tiangen Biotech, Beijing, China). A FastQuant RT Kit was utilized to reverse-transcribe total RNA (1.5 µg) into cDNA. SuperReal PreMix Plus (SYBR Green) was used for qPCR amplification using a Bio-Rad CFX 96 PCR system (Bio-Rad, USA). Actin was used for normalization. The qRT-PCR primers were provided by Sangon Biotech (Shanghai, China) [19].

### Volatile compound analyses

Volatile compounds were analyzed by gas chromatography-ion mobility spectrometry (GC-IMS). Before the analysis, 0.5 g of pepper fruit was placed in a 20-mL headspace container and incubated at 60℃ for 20 min. The NIST and IMS databases were used for qualitative examination of the volatile compounds. To analyze each sample from a different angle, VOCAL and three plugins (Reporter, Gallery Plot, and Dynamic PCA) were employed [20].Additional file 1: Table S7 contains a list of the precise instrumental parameters.

### Statistical analysis

Graphs were drawn using GraphPad Prism version 8.0 and data were analyzed using SPSS 26.0. The metabolic data were analyzed with MetaboAnalyst, and Tukey’s test (P˂0.05) was employed to distinguish distinct treatments. Multivariate statistical analysis was carried out using SIMCA 13.0 software. Differential metabolites were also analyzed using MetaboAnalyst 4.0 (http://www.metaboanalyst.ca).

## Results

### Statistical analysis and screening of differential metabolites in pepper roots

**Fig. 1:**
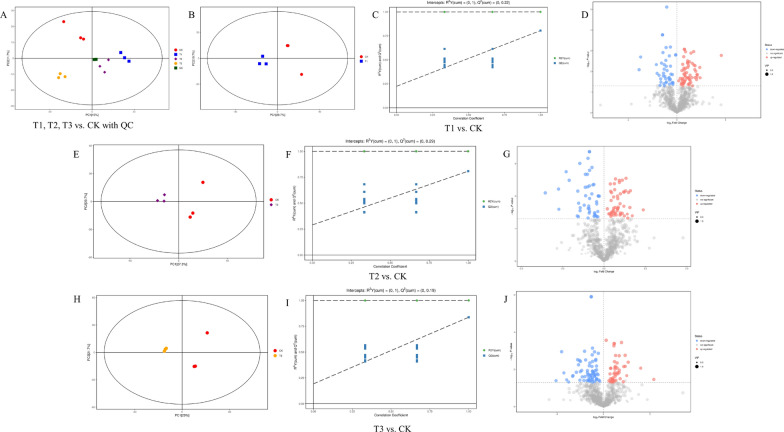
Effects of different nano-Se concentrations on metabolite concentrations in pepper root (**A**: T1, T2, T3 vs. CK; **B**, **C**, **D**: T1 vs. CK; **E**, **F**, **G**: T2 vs. CK; **H**, **I**, **J**: T3 vs. CK). **A**, **B**, **E**, **H**: principal component analysis (PCA); **C**, **F**, **I**: permutation plots of the validated models (PLS-DA); **D**, **G**, **J**: volcano plot; T1: nano-Se1; T2: nano-Se5; T3: nano-Se20

The EXION LC System (SCIEX) ultra-high performance liquid chromatography was used to analyze root metabolites after treatment with different nano-Se concentrations (0, 1, 5, and 20 mg/L) in Cd-contaminated soil (Fig. 1A–J). The types and contents of the metabolites and their related metabolic pathways in pepper roots were determined by wide-target metabolomics methods. PCA (Fig. 1A, B, E and H) showed that all samples were within the 95% confidence interval, and that there was a clear separation trend between the control and nano-Se treatments. The R^2^X, R^2^Y, and Q^2^ of the model parameter obtained through OPLS-DA modeling analysis and seven-fold cross-verification are shown in Additional file 1: Table S1.

**Fig. 2:**
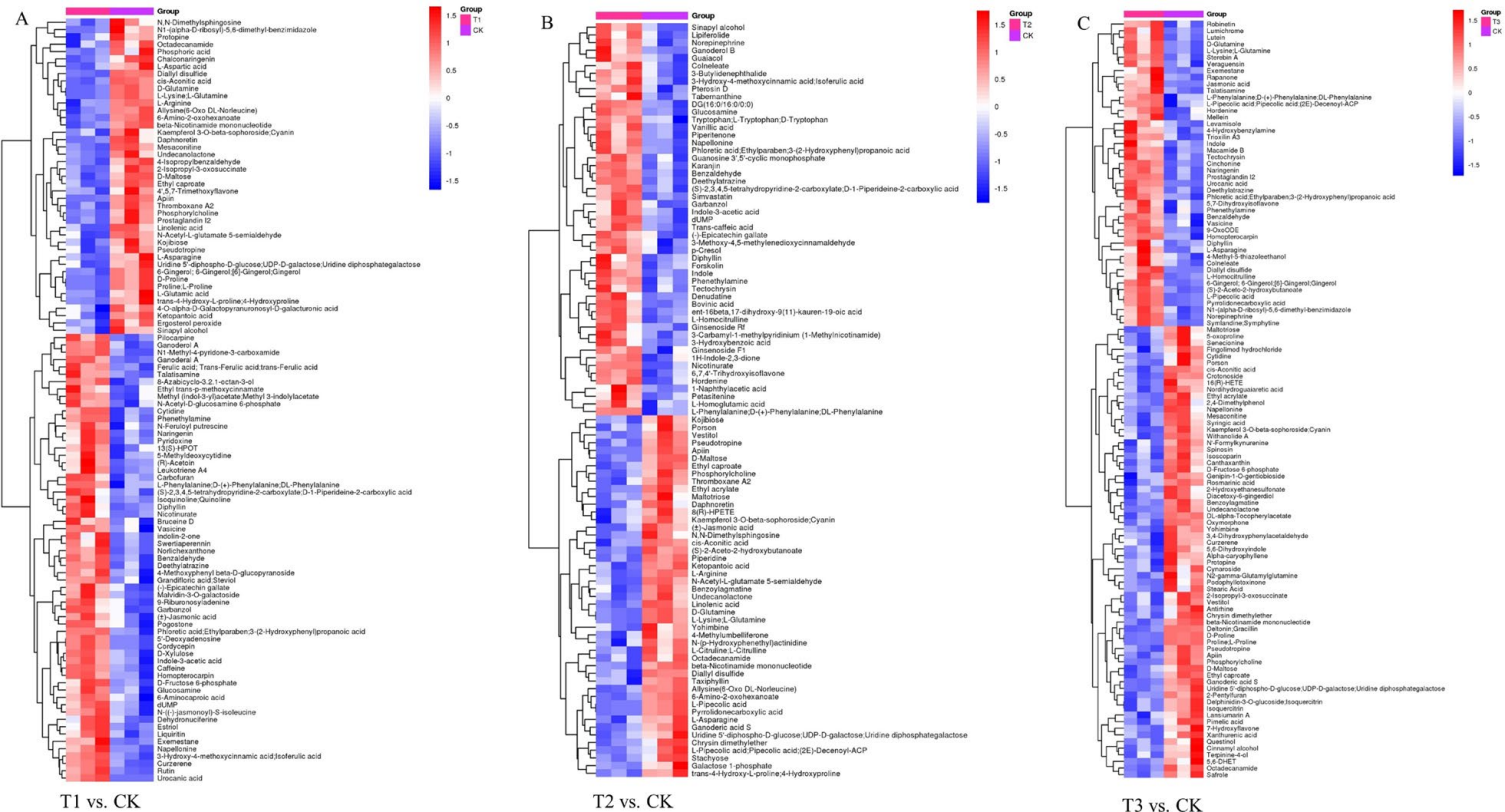
Heatmap of differential metabolites for pairwise comparison in pepper root: **A** T1 and CK; **B** T2 and CK; **C** T3 and CK. T1: nano-Se1; T2: nano-Se5; T3: nano-Se20

In this study, the P ; 0.05, FC < 0.5, or > 2 were chosen as the card values, and the VIP of the first principal component of the OPLS-DA model was ; 1. Different treatments (T1 vs. CK, T2 vs. CK, and T3 vs. CK) revealed 104, 98, and 114 metabolites, respectively (Additional file 1: Table S2). The metabolite types included amino acids, plant hormones, indoles and derivatives, flavonoids and phenolic acids, and pyridine and its derivatives. The selected differential compounds were evaluated visually using a volcano plot (Fig. 1D, G and J), with the size of the scatter representing the VIP value of the OPLS-DA model. In the figure, red indicates highly up-regulated metabolites, blue indicates significantly down-regulated metabolites, and gray indicates metabolites that did not differ significantly. The Euclidean distance matrix and complete linkage method were used for cluster analysis of the differential metabolites, shown by the heatmap of the analyzed differential metabolites (Fig. 2A, B and C).

### Analysis of differential metabolite pathways in pepper roots

**Fig. 3:**
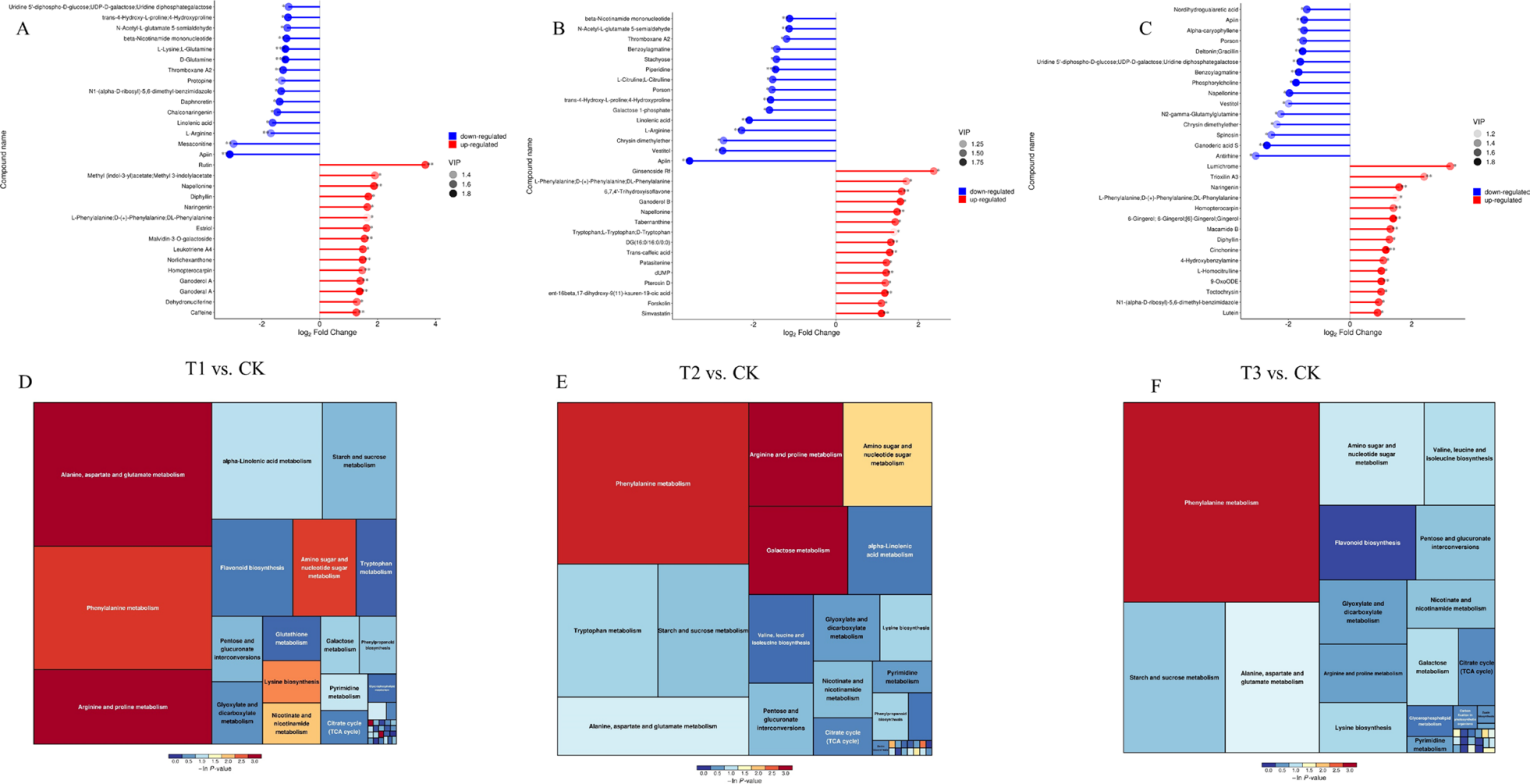
Matchstick and KEGG analyses of differential metabolites in pepper roots: **A**/**D** T1 and CK; **B**/**E** T2 and CK; **C**/**F** T3 and CK. T1: nano-Se1; T2: nano-Se5; T3: nano-Se20

For each group, the corresponding ratio of the quantitative value of the differential metabolites was computed, and the base 2 logarithm was applied with the horizontal coordinate reflecting the change multiple after logarithmic adjustment and the dot color representing the VIP value. These results showing the differential metabolites between the different treatments are shown as a matchstick diagram (Fig. 3A–C). The enrichment of the differential metabolites from the control and different nano-Se treatments was analyzed by KEGG to investigate their associated pathways. This showed that metabolites that differed between the T1 vs. CK treatments were mostly involved in alanine, aspartic acid, and glutamic acid metabolism, arginine and proline metabolism, and phenylpropanoid metabolism (Fig. 3D). Differential metabolites between the T2 vs. CK treatments were predominantly engaged in phenylpropane, arginine, and proline metabolism, as well as galactose metabolism (Fig. 3E) while metabolites that changed between the T3 and CK treatments were primarily involved in phenylpropane metabolism (Fig. 3F).

### Statistical analysis and screening of differential metabolites in pepper fruit

**Fig. 4:**
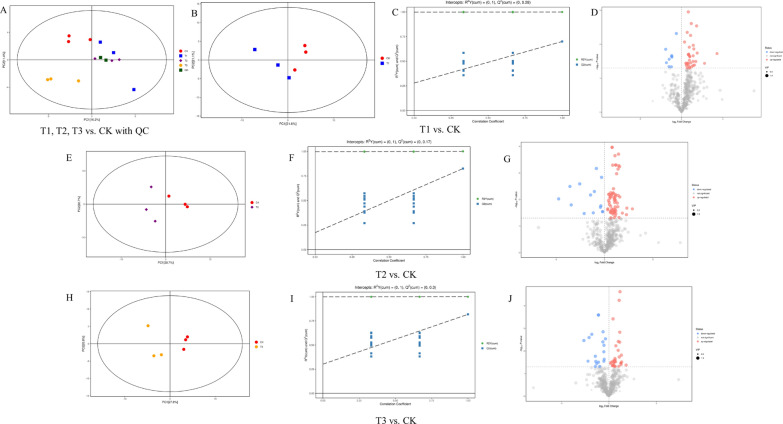
Effects of nano-Se on metabolite concentrations in pepper fruit (**A**: T1, T2, T3 vs. CK; **B**, **C**, **D**: T1 vs. CK; **E**, **F**, **G**: T2 vs. CK; **H**, **I**, **J**: T3 vs. CK). **A**, **B**, **E**, **H**: PCA; **C**, **F**, **I**: PLS-DA; **D**, **G**, **J**: volcano plot; T1: nano-Se1; T2: nano-Se5; T3: nano-Se20

Analysis of pepper fruit after treatment with different nano-Se concentrations (0, 1, 5, and 20 mg/L) was performed on an EXION LC System (SCIEX) for ultra-high-performance liquid chromatography (UHPLC) (Fig. 4A–J). Wide-target metabolomics was used to assess the types and concentrations of metabolites in pepper fruits, as well as the variations in the different metabolites and their associated metabolic pathways. PCA (Fig. 4A, B, E and H) revealed that the sample fell within the 95% confidence interval, and that there was clear separation between the control and nano-Se samples. The R^2^X, R^2^Y, and Q^2^ parameters (Additional file 1: Table S3) were examined using OPLS-DA modeling (7-fold cross-validation). Figure 4C, F, I illustrate the permutation test OPLS-DA model for the CK, T1, T2, and T3 groups.

**Fig. 5:**
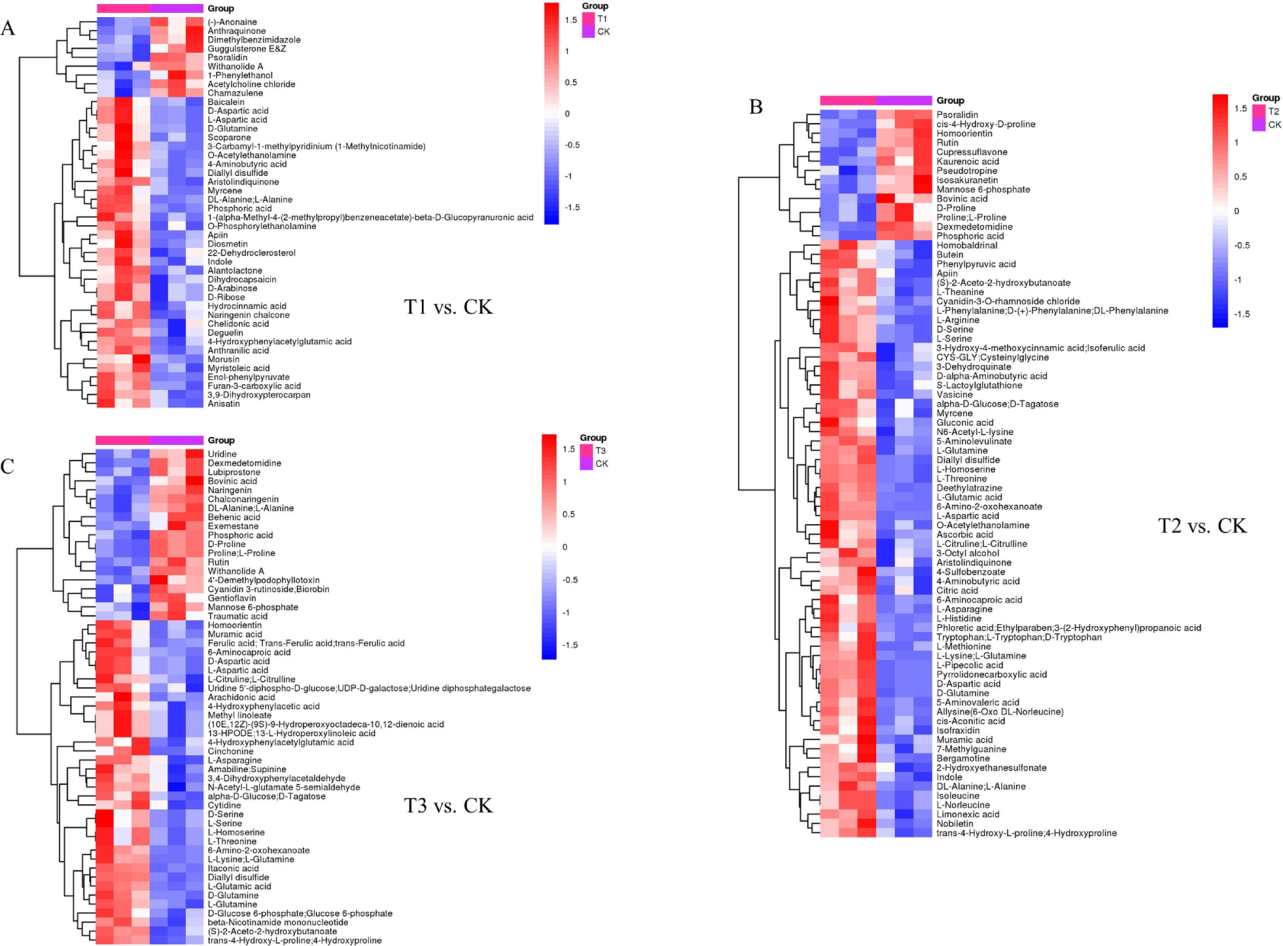
Heatmap of differential metabolites for pairwise comparison in pepper fruit: **A** T1 and CK; **B** T2 and CK; **C** T3 and CK. T1: nano-Se1; T2: nano-Se5; T3: nano-Se20

The card values used in this study were P < 0.05, FC < 0.5 or > 2, and the VIP of the first principal component of the OPLS-DA model was > 1. Totals of 44, 78, and 55 differential compounds were identified in the T1 vs. CK, T2 vs. CK, and T3 vs. CK groups, respectively (Additional file 1: Table S4). These metabolites were alkaloids, phenols, pyridines and derivatives, amino acids, and quinones. Visual analysis of the compounds is shown as volcano plots in Fig. 4D, G, and J. The full linkage approach and Euclidean distance matrix were then used for cluster analysis of the various compounds (Fig. 5A–C).

### Analysis of differential metabolite pathways in pepper fruit

**Fig. 6:**
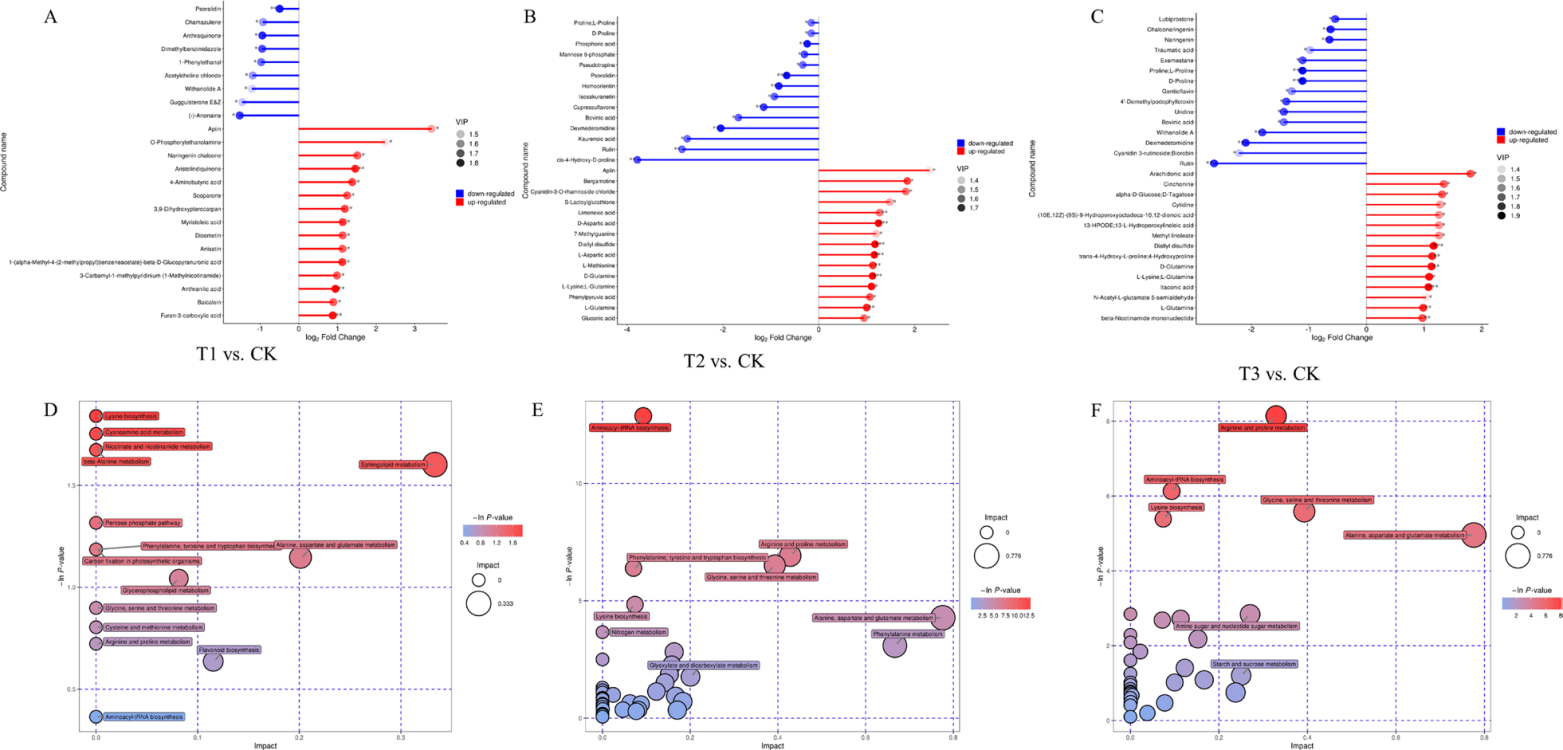
Matchstick and KEGG analyses of differential metabolites in pepper fruit: **A**/**D** T1 and CK; **B**/**E** T2 and CK; **C**/**F** T3 and CK. T1: nano-Se1; T2: nano-Se5; T3: nano-Se20

The logarithmic conversion with base 2 was used to obtain the quantitative ratios of the specific metabolites associated with each group. The horizontal coordinate represents the change multiple following the logarithmic translation, while the shade of the dot color indicates the VIP value. Metabolite changes in response to treatment were examined using matchstick diagrams. For each group of treatments, the top 15 down-regulated and up-regulated multiples of significant differences were selected for visual display in the matchstick diagram (Fig. 6A–C). In addition, KEGG pathway analysis of differential metabolites showed involvement in lysine biosynthesis, niacin and nicotinamide metabolism, β-alanine metabolism, sphingolipid metabolism (T1 vs. CK, Fig. 6D);aminoacyl-tRNA biosynthesis (T2 vs. CK, Fig. 6E); arginine and proline metabolism (T3 vs. CK, Fig. 6F).

### Changes in the amino acid distribution in pepper plants

**Fig. 7:**
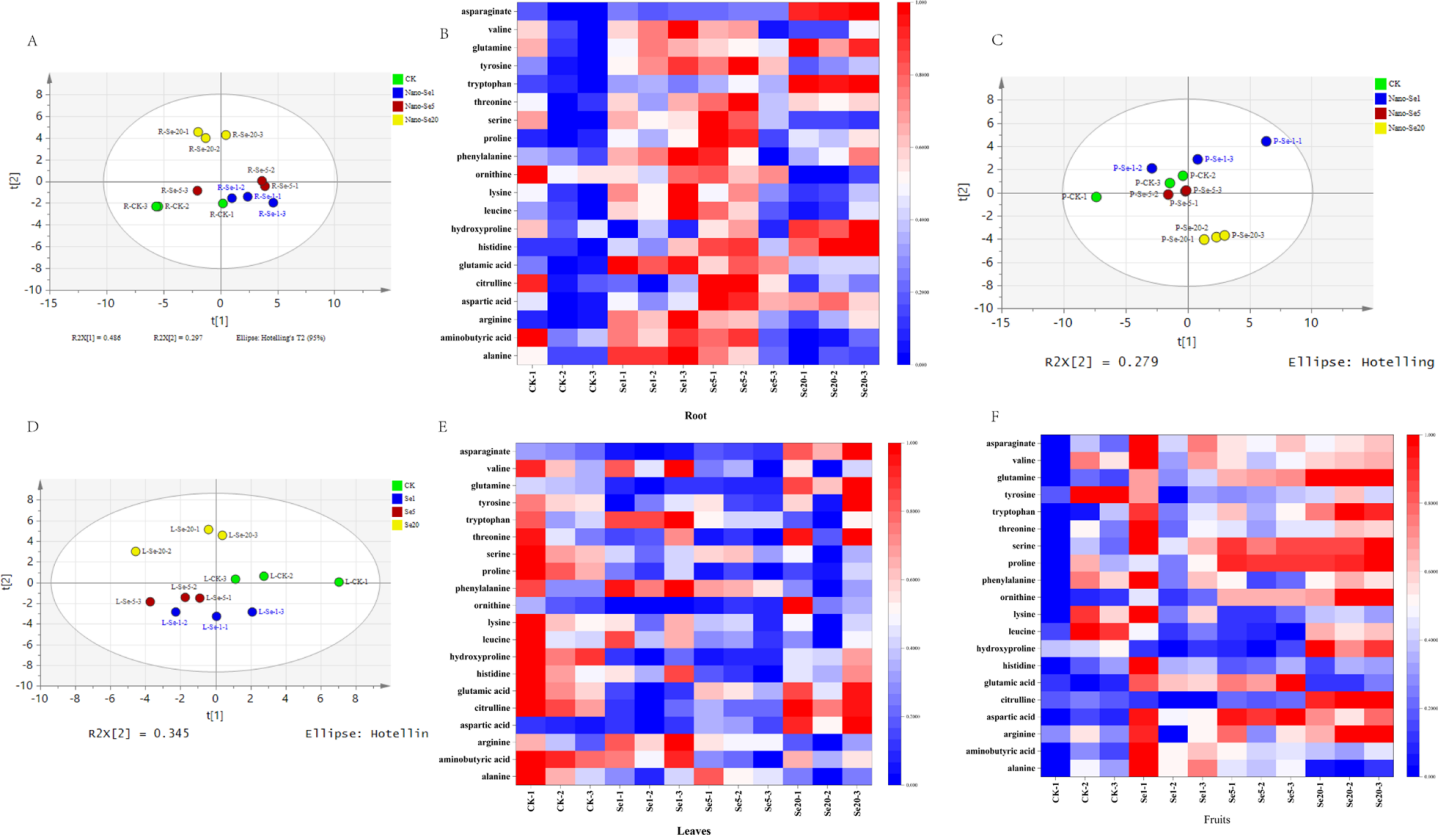
PCA and heatmap analysis of amino acids in pepper root, leaf, and fruit in the different treatment groups: **A**/**B** Pepper root; **D**/**E** Pepper leaves; **C**/**F** Pepper fruit

**Fig. 8:**
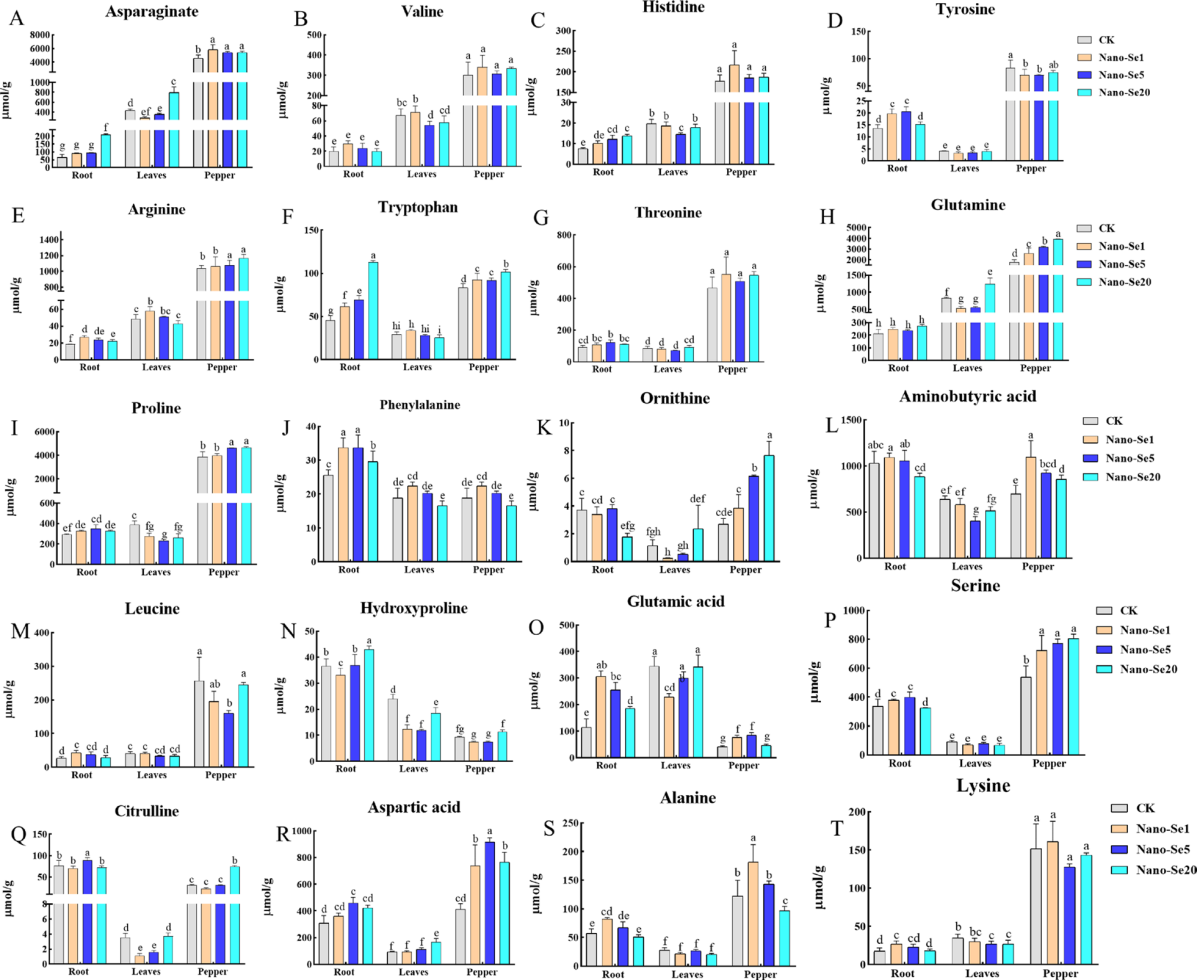
Effects of different concentrations of nano-Se (1, 5, and 20 mg/L) on the Asn (**A**), Val (B), His (**C**), Tyr (**D**), Arg (**E**), Trp (**F**), Thr (**G**), Gln (**H**), Pro (**I**), Phe (**J**), Orn (**K**), GABA (**L**), Leu (**M**), Hyp (**N**), Glu (**O**), Ser (**P**), Cit (**Q**), Asp (**R**), Ala (**S**), and Lys (**T**) levels in pepper plants (root, leaves, and fruit) under Cd stress. Significant differences at *P* < 0.05 are indicated by various letters for these treatments

The amino acid contents of pepper roots, leaves, and fruit in plants grown in Cd-contaminated soil and treated with nano-Se (0, 1, 5, and 20 mg/L) were determined (Figs. 7 and 8). The PCA results (Fig. 7A, C, and D) indicated that all treatment groups fell within the 95% confidence interval, with the nano-Se treatment group (1, 5, and 20 mg/L) separated from the control group. Cluster analysis of the specific metabolites is shown by heatmaps (Fig. 7B, E, and F). Under Cd stress, nano-Se considerably enhanced the amino acid levels in the roots compared with the control while leaves showed a dramatic reduction in amino acid levels that were increased again in the fruit when the nano-Se concentration was raised.

The aspartic acid (Asn), valine (Val), histidine (His), tyrosine (Tyr), arginine (Arg), tryptophan (Trp), threonine (Thr), glutamine (Gln), proline (Pro), phenylalanine (Phe), ornithine (Orn), aminobutyric acid (GABA), leucine (Leu), hydroxyproline (Hyp), glutamic acid (Glu), serine (Ser), citrulline (Cit), aspartic acid (Asp), alanine (Ala), and lysine (Lys) contents in the different plant organs are shown in Fig. 8A–T. Treatment with nano-Se did not significantly alter the Val, Thr, Lys, or Leu contents in any of the plant organs. In contrast, the levels of Tyr, Pro, Asp, and Ser in the roots first increased and then decreased as the nano-Se concentration increased (1, 5, and 20 mg/L), while His, Gln, Trp, and Hyp concentrations steadily increased and Phe, Arg, Glu, and Ala decreased markedly. Leaves showed marked reductions in the levels of hydroxyproline, citrulline, and ornithine (5 and 20 mg/L) and enhanced concentrations of Gln and Asp. In the fruit, there was a progressive increase in the Arg, Pro, Trp, Gln, Orn, Ser, Phe, and Cit contents in correspondence with increased nano-Se, while Gln and Asp first increased and subsequently declined, and the aminobutyric acid and Ala concentrations were gradually reduced.

### Changes in capsaicin-associated compounds and gene expression in pepper fruit

**Fig. 9:**
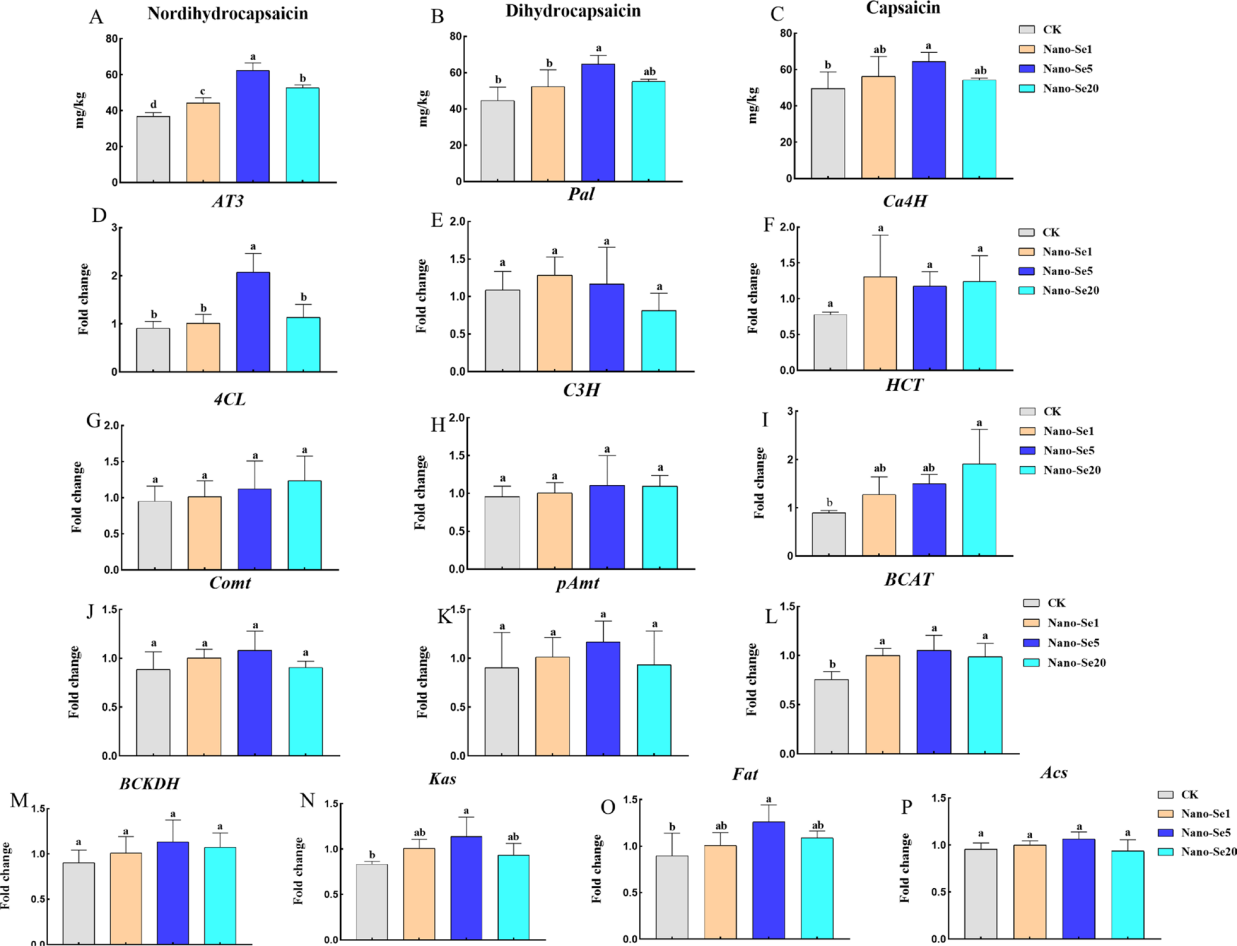
Effects of different concentrations of nano-Se on nordihydrocapsaicin **A**, dihydrocapsaicin **B**, capsaicin **C** contents, and capsaicinoid synthetic pathway-related gene **D–P** levels in pepper fruit. Different letters across treatments indicate significant differences at *P* < 0.05

As shown in Fig. 9, the levels of dihydrocapsaicin and nordihydrocapsaicin in pepper fruits increased initially and then declined as the nano-Se concentration increased, compared with the control. Nano-Se (1, 5, and 20 mg/L) enhanced the dihydrocapsaicin concentration by 20.4, 44.2, and 70.1%, respectively. Nano-Se5 enhanced the concentrations of capsaicin and dihydrocapsaicin by 29.6% and 45.3%, respectively.

The levels of the branched fatty acid (*BCDKH*, *BCAT*, *Kas*, *Acl*, *Acs*, and *Fat*), phenylpropane (*Pal*, *4CL*, *C3H*, *Ca4H*, *HCT*, *pAmt*, and *Comt*), and *AT3* genes were assessed by RT-PCR. Different concentrations of nano-Se had no appreciable impact on the expression of *Pal*, *Ca4H*, *4CL*, *C3H*, *Comt*, *pAmt*, *BCKDH*, and *Acs* when compared with the control. The levels of *AT3*, *Kas*, *Fat*, and *BCAT* first rose and then fell as the nano-Se concentration increased (1, 5, and 20 mg/L) while the expression of *HCT* steadily increased.

### Volatile organic compounds in pepper fruits

**Fig. 10:**
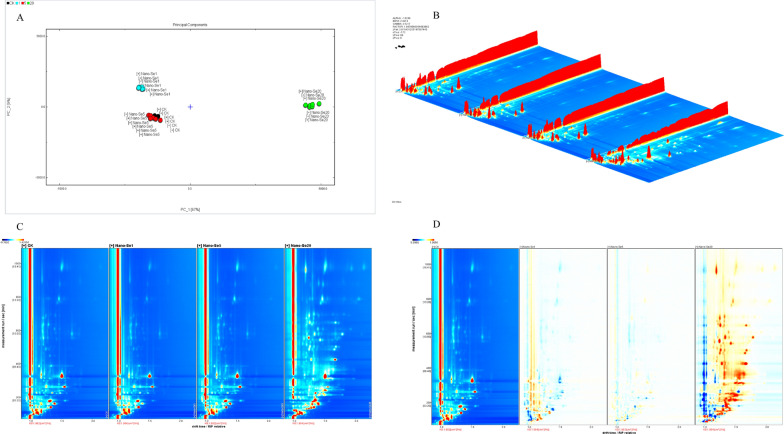
PCA (**A**), GC-IMS three-dimensional spectra (**B**), top view (**C**), and difference plot (**D**) of control and different nano-Se treatments

The flavor of pepper fruits treated with nano-Se at various concentrations was assessed by GC-IMS. Two main factors in the PCA (Fig. 10A) explained 96% of the variance (87% PC1 and 9% PC2) in pepper fruit. The separation of the data was more pronounced as the nano-Se concentration rose. Additionally, the variation in the volatile compounds may be reflected by the distance between samples. Therefore, the PCA used in this study could effectively distinguish the overall VOCs in pepper fruit treated with nano-Se.

Differences in the flavor compounds of pepper fruit treated with nano-Se can be readily compared by the projection of the GC-IMS spectrum onto the three/two-dimensional plane, as shown in Fig. 10B–D. The reactive ion peak is indicated by the red vertical line at abscissa 1.0 in the whole image’s blue backdrop (RIP peak). The ordinate represents the retention times in the gas chromatography, while the abscissa represents the ion migration time. Each point on either side of the RIP peak represents a VOC with the red color denoting high levels and white low levels. The volatile compounds have distinct spectral differences.

**Fig. 11:**
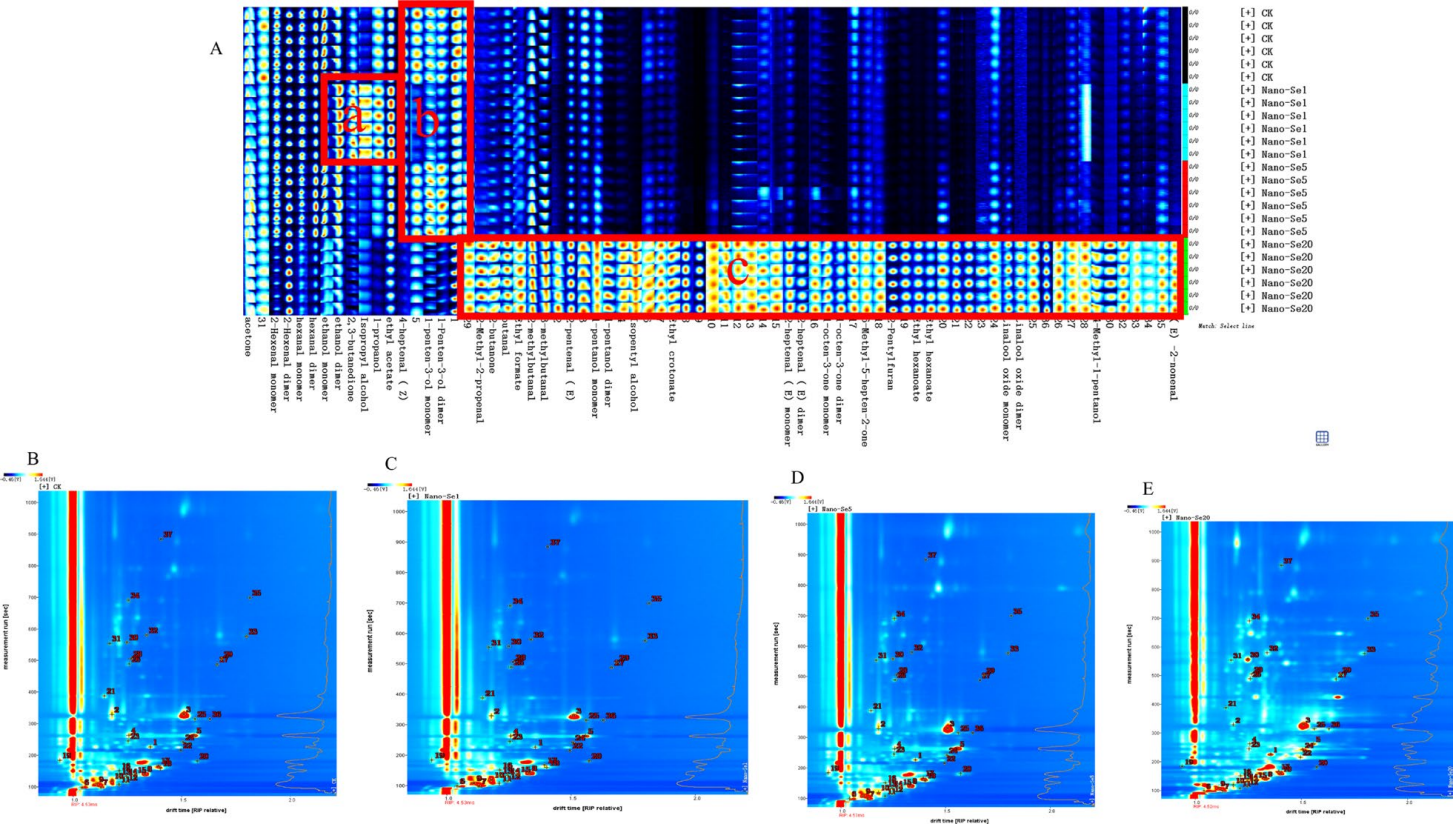
Gallery plot (**A**) and GC-IMS Library Search (**B–E**) of control and different nano-Se treatments. **B** CK; **C** nano-Se1; **D** nano-Se5; **E** nano-Se20

**Fig. 12:**
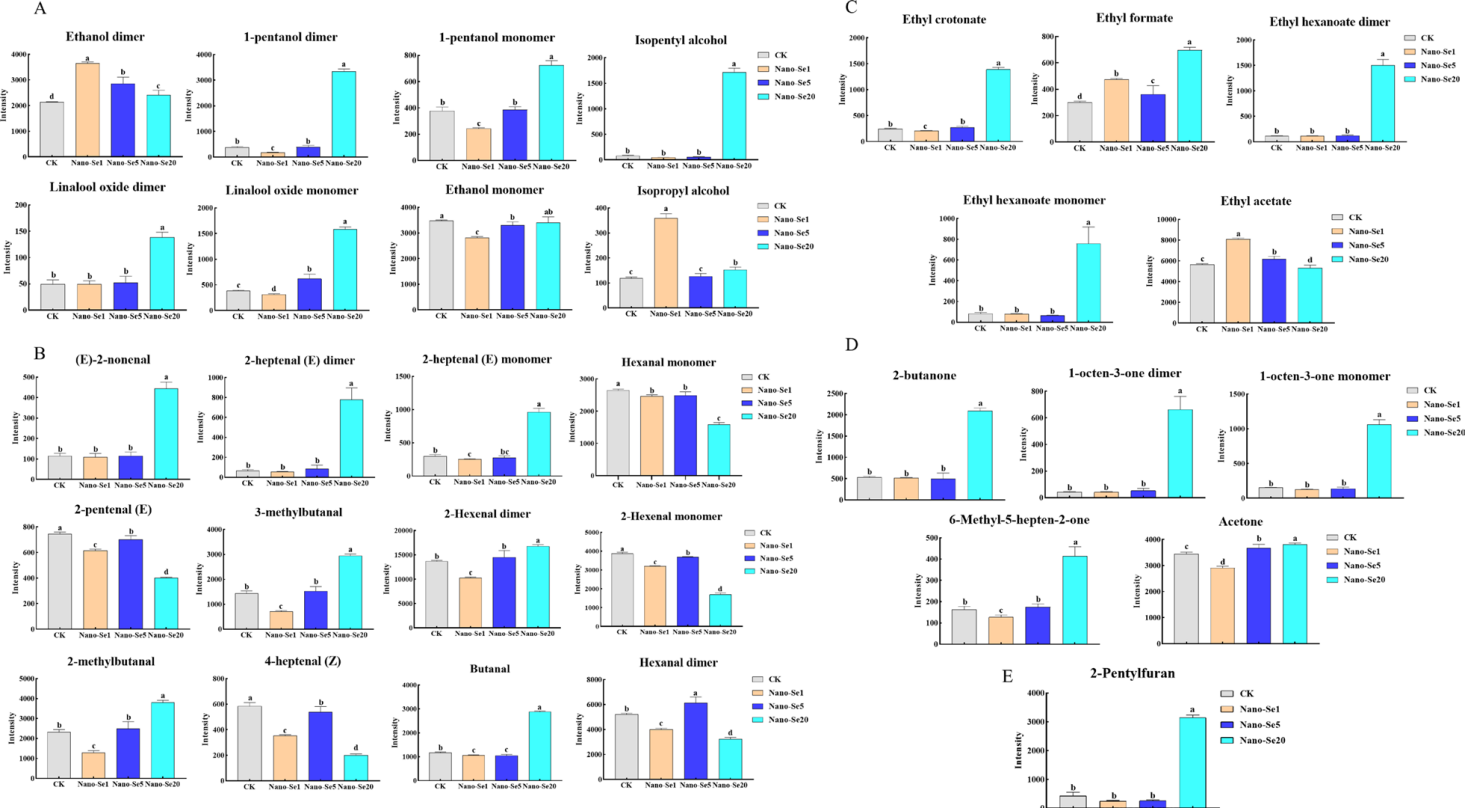
Changes in volatile compounds after nano-Se treatment in pepper fruit (**A**–**E**). Different letters across treatments indicate significant differences at *P* < 0.05. **A** alcohols; **B** aldehydes; **C** esters; **D** ketones; **E** furans

A qualitative analysis of the flavor substances was performed using a library search plug-in. Additional file 1: Table S5 shows detailed information on the volatile compounds shown in Fig. 11A. The identified volatile compounds had a variety of aroma components, including alcohols (12), aldehydes (13), esters (5), ketones (6), and furans (1). A more thorough evaluation of the variations in flavor compounds between the samples was performed using LAV software’s Gallery Plot plug-in, which automatically constructs the fingerprints of chromatographic peaks (Fig. 11B–E). The fingerprint obtained was compared with volatile chemicals found in various peppers. Figure 11 A shows the differences in volatile compounds in samples treated with different nano-Se concentrations. The fingerprint shows that nano-Se (20 mg/L) had both the greatest variety and greatest concentrations of volatile chemicals, which is the most noticeable difference between the samples. The chemical concentrations were mostly highest in nano-Se1, and were low or non-existent in other samples. The volatile chemicals were mostly ethanol, 2, 3-butanedione, isopropyl alcohol, n-propyl alcohol, and ethyl acetate, as shown from left to right (Fig. 11A). From left to right, the concentration of chemicals in area b is greater in CK and nano-Se5 than in other samples, primarily due to the presence of Z-4-heptanal and 1-pentene-3-ol. In addition, substances in area c showed the greatest concentrations in the nano-Se20 sample, with low or absent concentrations in the other samples. From left to right, these are 2-methyl-2-propionaldehyde, 2-butanone, ethyl formate, 2-methyl-butanaldehyde, 3-methyl-butanaldehyde, E-2-heptanaldehyde, amyl alcohol, isoamyl alcohol, ethyl crotonate, E-2-heptanaldehyde, 1-octene-3-one, 6-methyl-5-heptene-2-one, 2-amyl furan, ethyl caproate, linalool oxide, 4-methyl-1-pentanol, and E-2-nonaldehyde. Figure 12A–E summarizes the effects of nano-Se application on the volatile compound composition of pepper.

## Discussion

In crops, Cd toxicity reduces nutrient and water uptake, increases oxidative damage, disrupts plant metabolism, and adversely affects both plant morphology and physiology. There is limited information on the changes in crop nutrients and their associated pathways in response to Cd stress, particularly, the concentrations of VOCs, compared with physiological and biochemical indicators. It is critical to boost pepper plants’ nutritional value and tolerance to Cd contamination in the soil. Our earlier research demonstrated that nano-Se can promote the growth and development of pepper plants under Cd stress, improving the soil properties and the distribution of relevant signaling molecules, as well as increasing the levels of primary and secondary metabolites and reducing both Cd uptake and toxicity [16]. However, there is no information on the pepper plant’s general metabolic level, fruit nutrients, or volatile organic chemicals. In this study, it was found that nano-Se could improve the plant’s Cd tolerance by regulating the amino acid-related resistance pathways. This also resulted in the modulation of the phenylpropane and branched fatty acid pathways, as well as increasing the levels of capsaicin-associated compounds and greatly improving the composition and amounts of VOCs in the fruit.

Importantly, soil irrigation with nano-Se (1, 5, and 20 mg/L) increased the levels of dihydrocapsaicin and nordihydrocapsaicin to varying degrees under Cd stress but had no discernible impact on the content of capsaicin (Fig. 9A–C). Additionally, the contents of genes involved in capsaicin syntheses, such as *HCT*, *AT3*, *Kas*, *Fat*, and *BCAT*, were significantly increased by nano-Se treatment (Fig. 9D–P). According to our previous findings, foliar spraying of nano-Se5 and nano-Se20 regulates phenylpropanoid metabolic pathways (*Pal*, *4CL*, *HCT*, and *pAmt*), branched fatty acid pathways (*Kas*, *Acl*, and *Fat*), and expression of the key *AT3* gene, thus significantly increasing the synthesis of capsaicin and dihydrocapsaicin [19]. This may be connected to how the Se is applied. Many variables, including plant species, soil, environment, and nanoscale characteristics, determine how Se affects plants. Se enhances plant nutrient contents by efficient absorption through the leaves as the leaf plasmodesmata have nanopores that facilitate the simple usage of chemicals [21]. Foliar spraying of Se was also found to result in greater grain production in comparison with soil application, indicating that selenium application on leaves is more easily transported through the phloem [22]. In some cases, foliar spraying of Se may have little impact on metal absorption. Se addition to soil may be more effective in reducing heavy metal concentrations in plant tissues than foliar spraying according to currently available data. Wu et al. showed that the application of Se (Na_2_SeO_3_) alone on the leaf surface did not significantly reduce the concentration of Cd in root cell walls and branches of Chinese cabbage [23] while Hussain et al. found that foliar spraying of nano-Se (20 mg/L) did not significantly lower Cd and Pb levels in rice [24]. Additionally, pot experiments demonstrated that in comparison with leaf treatment, soil Se (IV) and Se (VI) considerably decreased the Hg concentrations in rice tissues [25]. Therefore, additional research should be conducted to assess the effectiveness of different Se treatments in reducing heavy metal concentrations in plants together with elucidating the regulatory mechanisms controlling nutrient distribution in crops.

Crop nitrogen metabolism is known to be impacted by the stress of Cd-contaminated soil, altering amino acid concentration and amino acid-related pathways. Amino acids are the components and precursors of proteins, and changes in their metabolism can alter enzyme activity, gene expression, redox homeostasis, and ion transport regulation, amongst other processes. Plants play crucial roles in the adaptation of ecosystems to stress [26]. Xu et al. observed significant decreases in amino acid contents in response to increased Cd concentrations in rice grains. The contents of eight amino acids (Glu, Phe, Arg, His, Lys, Ser, Ala, and Thr) in grains were found to be significantly correlated with the Cd content [26]. Ulhassan et al. reported that Se enhanced amino acid metabolism/biosynthesis and alleviated oxidative stress by lowering Cr-induced amino acids concentrations (Leu, Val, Lys, Thr, Phe), counteracting Cr-induced damage in rapeseed [27]. Our study found that the differential metabolites in roots were mostly engaged in alanine, aspartic acid, glutamic acid metabolism, arginine, proline metabolism, and phenylpropane metabolism through wide-target metabolomics (Fig. 3). Figure 8 indicates that nano-Se biofortification significantly raised the levels of Phe, Asp, Glu, Arg, and Pro. This study also discovered that the primary pathways involved in different compounds in pepper fruit include β-alanine metabolism, aminoacyl-tRNA biosynthesis, Arg and Pro metabolism (Fig. 6). Different nano-Se concentrations can enhance the amino acid concentration in the root (Pro, Arg, His, Gln, Trp, Hyp, Tyr, Ser, and Ala) and fruit (Pro, Arg, Trp, Gln, Orn, Ser, Phe, Cit, Glu, Asp, GABA, and Ala) shown by target verification (Fig. 8A–T). Previous studies also found that foliar spraying of nano-Se (5 and 20 mg/L) could regulate the proline pathway and increase the Pro content in pepper fruit [19]. Under heavy metal stress, increased production of Pro in plant cells contributes to the preservation of cellular homeostasis, water absorption, osmotic regulation, and redox balance, to repair cell structure and reduce oxidative damage [28]. Glu also plays a key role in nutrition, metabolism, and signaling. Glu is used as an amino donor in most trans-aminogenic processes in plants, including the biosynthesis of amino acids such as Gln, GABA, Arg, Pro, Gly, Asp, Ala, Ser, Phe, Tyr, and His [29]. Flavonoids from Phe or Tyr accumulate significantly under various abiotic stress conditions such as ultraviolet light, temperature, salt, heavy metal, and drought stress [30, 31]. Polyamines are synthesized by Arg, Orn, and Cit, and are greatly increased during abiotic stress. Overexpression of enzymes involved in the polyamine pathway leads to higher stress tolerance, implying a protective effect [32]. Other amino acids, including GABA [33], His [32], Trp [34], Asp [35], Orn [36], and Ala [37] are also strongly associated with abiotic stress (cold injury, salt, drought, and heavy metal stress, as well as nutritional quality) [38]. Thus, Se can either directly or indirectly boost the synthesis of amino acids and trigger the production of a variety of primary and secondary metabolites (fatty acids and their oxidation products, antioxidants, glucosinolate, and phenolic compounds) [39].

Moreover, nano-Se biofortification may dramatically improve the VOC content of pepper fruit, thus increasing its nutritional value. Among the secreted metabolites, VOCs have been shown to induce plant immunity when applied to plants. Previous studies have shown that a combination of nano-Se (5 mg/L) and melatonin (10 mg/L) treatment improved the VOCs of insect attractants and repellents in wheat, such as ethanol, 1-decanol, isoamyl alcohol, 2-butanone, 2-heptanone, acetone, benzaldehyde, ethyl 2-methylpropionate, and ethyl acetate dimer [40]. In this study (Fig. 12A–E), increased nano-Se concentrations led to significant increases in the levels of alcohols (amyl alcohol monomer and dimer, isoamyl alcohol, oxidation of aromatic camphor alcohol monomer and dimer), aldehydes (E-2-nonyl aldehyde, E-2-heptyl aldehyde, 3-methyl butyl aldehyde, 2-hexene aldehyde monomer, and 2-methyl butyl aldehyde), esters (crotonic acid ethyl ester, ethyl caproate and ethyl formate), ketones (2-butanone, 1-octene-ketone of 3-,6-Methyl-5-heptene-2-one, acetone), and furans (2-amyl furan). Rudell et al. observed that amyl alcohol dramatically increased the contents of 1-butanol, 2-methyl-1-butanol, and 1-hexanol in pepper fruit tissues under hypoxia [41] while Zahir et al. found that adding adenine to isopentyl alcohol raised the plant height, tiller number, panicle number, rice yield and nitrogen, phosphorus and potassium contents of straw and grains [42]. Aromatic alcohol oxides are not only the principal aroma components of crops such as tea [43], carnation [44], and fruit [45], but they also act as synergists to improve the trapping of sex pheromones in insects (*Sophora officinalis*) [46]. Among the aldehydes, trans-2-hexenal can inhibit the normal physiological and biochemical activities of nematodes from different crops [47, 48]; the incidence of botrytis cinerea in tomatoes treated with trans-2-hexenal was dramatically reduced [49](E) -2-hexenal plus methyl jasmonate (MeJA) boosted anthocyanin content in *Arabidopsis thaliana* [50]. Meucci et al. discovered that foliar spraying of Se (VI) increased VOCs, including 2-phenylethanol, guacacol, (E) -2-hexenal, 1-pentene-3-ketone, and (E)-2-pentenal, which were positively correlated with consumer preference and taste intensity [51]. Caitlin et al. showed that (Z)-3-hexenol, isobutyral, 2-methyl butyral, and 3-methyl butyral emissions of buckwheat were higher under drought stress, which had a substantial impact on floral characteristics and pollinator attractiveness [52]. It was determined that combining ethyl formate and phosphine enhanced the toxicity of ester compounds against the cotton bollworm [53]. Among the ketones, Song et al. showed that the usage of volatile organic compounds 3-amyl alcohol and 2-butanone boosted fruit yield and markedly increased ladybug populations, which are natural enemies of aphids [54]. (E)-orange alcohol and (E)-β-caryophyllene in maize and 6-methyl-5-heptene-2-one in soybean may also affect host plant selection by aphids and habitat search in lady beetles [55]. Lazazzara et al. observed that 6-amyl-2 H-pyrane-2-one and 2-amyl-furan increased callose production and promoted the regulation of defense-related genes in VOCs induced by grape mildew frost disease [56].

## Conclusion

In this study, we investigated the effects and mechanism of selenium nanoparticles in regulating primary metabolism and related resistance pathways involved in capsaicin synthesis, as well as VOCs in pepper fruit under Cd stress. The roots and fruit of pepper plants treated with Se were shown by wide-target metabolomics analysis to be engaged in key arginine and proline-associated metabolic pathways. It was found that the amino acid concentrations in roots (Pro, Gln, Trp, Arg, and Hyp,) and fruit (Pro, Arg, Trp, Gln, Orn, Phe, Glu, Asp, and GABA) were significantly increased after nano-Se treatment. The treatment also significantly improved the production of the capsaicin compounds (dihydrocapsaicin and nordihydrocapsaicin) as well as the expression of genes associated with capsaicin synthesis (*HCT*, *AT3*, *Kas*, *Fat*, and *BCAT*). It was also found that the contents of a substantial number of VOCs increased in response to increased nano-Se concentrations. These included alcohols (amyl alcohol and aromatic alcohol oxides), aldehydes (3-methyl-butanal, 2-hexenal, and 2-methyl-butanal), esters (ethyl formate), ketones (2-butanone and 6-methyl-5-heptene-2-one), and furans (2-amyl furan). Significant correlations between these VOCs and plant quality and resistance were found. In conclusion, nano-Se can improve fruit quality and health by regulating the amino acid levels of pepper plants, boosting the plant’s resistance, and stimulating the synthesis of capsaicin-associated compounds and VOCs.

## Supplementary Information

**Additional file 1:  Table S1.** OPLS-DA model reference table in pepper roots; **Table S2**. Differential metabolites were identified in pepper roots treated with control and different concentrations of nano-Se; **Table S3**. OPLS-DA model reference table in pepper fruit; **Table S4**. Differential metabolites were identified in pepper fruit treated with different concentrations of nano-Se; **Table S5**. Qualitative identification of compounds in gas phase ion migration spectra; **Table S6**. Instrumental parameters of three capsaicin compounds in pepper fruit; **Table S7**. Analysis conditions of the GC-IMS system.

## Data Availability

All data generated or analyzed during this study are included in this published article within each editable graph.
