# Supplementary material for: Metabolomic analysis on the mechanism of nanoselenium alleviating cadmium stress and improving the pepper nutritional value
Source: J Nanobiotechnology. 2022 Dec 10;20:523. doi: 10.1186/s12951-022-01739-5 (PMC9741789; doi:10.1186/s12951-022-01739-5)
Supplement: Supplementary file 1 — Additional file 1: Table S1. OPLS-DA model reference table in pepper roots; Table S2. Differential metabolites were identified in pepper roots treated with control and different concentrations of nano-Se; Table S3. OPLS-DA model reference table in pepper fruit; Table S4. Differential metabolites were identified in pepper fruit treated with different concentrations of nano-Se; Table S5. Qualitative identification of compounds in gas phase ion migration spectra; Table S6. Instrumental parameters of three capsaicin compounds in pepper fruit; Table S7. Analysis conditions of the GC-IMS system. [file 12951_2022_1739_MOESM1_ESM.docx]

Metabolomic analysis on the mechanism of nanoselenium alleviating cadmium stress and improving the pepper nutritional value

Dong Lib 1, Chunran Zhoua 1, Jia-Qi Li a c, Qinyong Donga c, Peijuan Miao a, Yongxi Lin a, Haiyan Chenga , Yuwei Wanga , Luna Luoa , Canping Pana,[*](#_bookmark7)

a Innovation Center of Pesticide Research, Department of Applied Chemistry, College of Science, China Agricultural University, Beijing 100193, China

^b^ Key Laboratory of Green Prevention and Control of Tropical Plant Diseases and Pests, Ministry of Education, College of Plant Protection, Hainan University, Haikou, Hainan 570228, People’s Republic of China

^c^ Key Laboratory of Tropical Fruits and Vegetables Quality and Safety for State Market Regulation, Haikou 570311, China

Canping Pan, canpingp@cau.edu.cn

1 Dong Li and Chunran Zhou contributed equally to this paper.

Total number of pages: 36

Total number of tables: 7

**SUPPORTING TABLES**

**Table S1.** OPLS-DA model reference table in pepper roots

**Table S2.** Differential metabolites were identified in pepper roots treated with control and different concentrations of nano-Se

**Table S3.** OPLS-DA model reference table in pepper fruit

**Table S4.** Differential metabolites were identified in pepper fruit treated with different concentrations of nano-Se

**Table S5.** Qualitative identification of compounds in gas phase ion migration spectra

**Table S6.** Instrumental parameters of three capsaicin compounds in pepper fruit

**Table S7.** Analysis conditions of the GC-IMS system

**Table S1. OPLS-DA model reference table in pepper roots**

| Type | N | R^2^X | R^2^Y | Q^2^ | Title |
| --- | --- | --- | --- | --- | --- |
| OPLS-DA | 6 | 0.49 | 1 | 0.805 | T1 vs CK |
| OPLS-DA | 6 | 0.472 | 1 | 0.808 | T2 vs CK |
| OPLS-DA | 6 | 0.506 | 1 | 0.837 | T3 vs CK |

**Table S2.** **Differential metabolites were identified in pepper roots treated with control and different concentrations of nano-Se**

| Differential metabolites of nano-selenium (1 mg/L) and control in pepper root | | | | | | | | | | | | |
| --- | --- | --- | --- | --- | --- | --- | --- | --- | --- | --- | --- | --- |
| Number | Compounds | Q1 | Q3 | RT | Mean T1 | Mean CK | VIP | P-Value | Q- Value | Fold Change | Log_Fold Change |  |
| 1 | (-)-Epicatechin gallate | 443.1 | 123 | 6.20 | 1.20E-03 | 8.86E-04 | 1.60 | 0.03 | 0.24 | 1.36 | 0.44 |  |
| 2 | (±)-Jasmonic acid | 209 | 59 | 8.94 | 6.46E-04 | 5.81E-04 | 1.59 | 0.03 | 0.24 | 1.11 | 0.15 |  |
| 3 | (R)-acetoin | 89.1 | 71 | 0.53 | 7.10E-05 | 5.88E-05 | 1.67 | 0.02 | 0.21 | 1.21 | 0.27 |  |
| 4 | (S)-2,3,4,5-tetrahydropyridine-2-carboxylate | 128.1 | 82.1 | 1.74 | 1.40E-04 | 6.62E-05 | 1.63 | 0.03 | 0.23 | 2.11 | 1.08 |  |
| 5 | 13(s)-hpot | 311.2 | 69.1 | 11.47 | 2.63E-04 | 1.71E-04 | 1.57 | 0.03 | 0.24 | 1.54 | 0.62 |  |
| 6 | 2-Isopropyl-3-oxosuccinate | 175.1 | 129.1 | 0.80 | 9.88E-05 | 1.38E-04 | 1.71 | 0.01 | 0.17 | 0.71 | -0.49 |  |
| 7 | 3-Hydroxy-4-methoxycinnamic acid | 195.1 | 149.1 | 4.87 | 1.53E-04 | 6.88E-05 | 1.77 | 0.00 | 0.07 | 2.23 | 1.15 |  |
| 8 | 4-isopropylbenzaldehyde | 149.1 | 43.1 | 10.56 | 7.05E-06 | 1.02E-05 | 1.76 | 0.01 | 0.12 | 0.69 | -0.53 |  |
| 9 | 4-Methoxyphenyl beta-D-glucopyranoside | 287.1 | 125.1 | 5.52 | 2.33E-05 | 1.29E-05 | 1.56 | 0.02 | 0.20 | 1.81 | 0.85 |  |
| 10 | 4-O-alpha-D-Galactopyranuronosyl-D-galacturonic acid | 353.1 | 97 | 11.43 | 9.00E-05 | 1.83E-04 | 1.33 | 0.05 | 0.30 | 0.49 | -1.03 |  |
| 11 | 4',5,7-trimethoxyflavone | 329.1 | 107 | 13.87 | 5.91E-06 | 1.11E-05 | 1.59 | 0.04 | 0.26 | 0.53 | -0.90 |  |
| 12 | 5-methyldeoxycytidine | 242.1 | 126.1 | 0.54 | 8.07E-06 | 3.95E-06 | 1.51 | 0.04 | 0.27 | 2.04 | 1.03 |  |
| 13 | 5'-deoxyadenosine | 252.1 | 136.1 | 2.41 | 1.18E-03 | 7.73E-04 | 1.82 | 0.00 | 0.07 | 1.53 | 0.62 |  |
| 14 | 6-Amino-2-oxohexanoate | 146.1 | 72.1 | 0.77 | 4.36E-03 | 8.08E-03 | 1.82 | 0.00 | 0.07 | 0.54 | -0.89 |  |
| 15 | 6-Aminocaproic acid | 132.1 | 41 | 1.84 | 2.23E-03 | 1.71E-03 | 1.51 | 0.04 | 0.27 | 1.30 | 0.38 |  |
| 16 | 6-gingerol | 293.2 | 57 | 11.39 | 1.56E-05 | 2.92E-05 | 1.79 | 0.00 | 0.09 | 0.53 | -0.91 |  |
| 17 | 8-Azabicyclo-3.2.1-octan-3-ol | 128.1 | 110.1 | 14.09 | 1.33E-05 | 8.10E-06 | 1.69 | 0.01 | 0.13 | 1.64 | 0.72 |  |
| 18 | 9-riburonosyladenine | 282.1 | 136.1 | 12.54 | 2.82E-05 | 1.27E-05 | 1.52 | 0.03 | 0.26 | 2.21 | 1.15 |  |
| 19 | Allysine(6-oxo dl-norleucine) | 146.1 | 100.1 | 0.77 | 3.17E-02 | 4.87E-02 | 1.78 | 0.00 | 0.08 | 0.65 | -0.62 |  |
| 20 | Apiin | 565.5 | 432.9 | 6.46 | 1.05E-04 | 9.03E-04 | 1.86 | 0.01 | 0.14 | 0.12 | -3.11 |  |
| 21 | Benzaldehyde | 107 | 79 | 2.82 | 3.20E-04 | 1.65E-04 | 1.71 | 0.01 | 0.11 | 1.94 | 0.95 |  |
| 22 | Beta-Nicotinamide mononucleotide | 335.1 | 123.1 | 0.73 | 3.12E-04 | 6.91E-04 | 1.79 | 0.00 | 0.07 | 0.45 | -1.15 |  |
| 23 | Bruceine D | 411.2 | 393.2 | 4.84 | 2.56E-05 | 1.35E-05 | 1.48 | 0.04 | 0.28 | 1.89 | 0.92 |  |
| 24 | Caffeine | 195.1 | 138.1 | 4.50 | 1.22E-04 | 5.07E-05 | 1.65 | 0.00 | 0.11 | 2.40 | 1.26 |  |
| 25 | Carbofuran | 222.1 | 123 | 4.03 | 3.60E-05 | 1.70E-05 | 1.63 | 0.02 | 0.18 | 2.12 | 1.08 |  |
| 26 | Chalconaringenin | 272.9 | 152.9 | 8.78 | 5.03E-06 | 1.39E-05 | 1.69 | 0.02 | 0.18 | 0.36 | -1.46 |  |
| 27 | Cis-Aconitic acid | 175 | 69 | 1.46 | 8.26E-03 | 1.19E-02 | 1.82 | 0.00 | 0.07 | 0.69 | -0.53 |  |
| 28 | Cordycepin | 252.1 | 136.1 | 2.35 | 1.18E-03 | 7.73E-04 | 1.82 | 0.00 | 0.07 | 1.53 | 0.62 |  |
| 29 | Curzerene | 217.2 | 55.1 | 2.55 | 1.08E-04 | 7.33E-05 | 1.80 | 0.00 | 0.08 | 1.48 | 0.56 |  |
| 30 | Cytidine | 244.1 | 112.1 | 0.70 | 1.51E-04 | 1.09E-04 | 1.75 | 0.00 | 0.11 | 1.39 | 0.48 |  |
| 31 | D-Fructose 6-phosphate | 259 | 97 | 3.23 | 3.01E-05 | 1.91E-05 | 1.68 | 0.02 | 0.18 | 1.58 | 0.66 |  |
| 32 | D-glutamine | 147.1 | 84 | 0.66 | 5.09E-03 | 1.16E-02 | 1.85 | 0.00 | 0.04 | 0.44 | -1.18 |  |
| 33 | D-maltose | 341.1 | 59 | 0.76 | 1.78E-02 | 3.26E-02 | 1.74 | 0.01 | 0.12 | 0.55 | -0.87 |  |
| 34 | D-proline | 116.1 | 70.1 | 0.76 | 2.99E-02 | 3.20E-02 | 1.81 | 0.00 | 0.08 | 0.94 | -0.10 |  |
| 35 | D-xylulose | 133.1 | 43 | 1.76 | 1.95E-04 | 1.46E-04 | 1.69 | 0.01 | 0.14 | 1.33 | 0.41 |  |
| 36 | Daphnoretin | 353.1 | 163 | 11.45 | 2.01E-04 | 5.26E-04 | 1.76 | 0.05 | 0.29 | 0.38 | -1.38 |  |
| 37 | Deethylatrazine | 188.1 | 146 | 3.81 | 1.18E-02 | 7.96E-03 | 1.71 | 0.01 | 0.13 | 1.49 | 0.57 |  |
| 38 | Dehydronuciferine | 294.1 | 206.1 | 5.66 | 2.15E-05 | 8.84E-06 | 1.53 | 0.05 | 0.30 | 2.43 | 1.28 |  |
| 39 | Diallyl disulfide | 147 | 41 | 0.68 | 3.49E-04 | 6.22E-04 | 1.86 | 0.00 | 0.01 | 0.56 | -0.83 |  |
| 40 | Diphyllin | 381.1 | 335.1 | 10.09 | 6.71E-06 | 2.09E-06 | 1.74 | 0.01 | 0.18 | 3.20 | 1.68 |  |
| 41 | Dump | 309 | 113 | 0.76 | 2.81E-05 | 1.43E-05 | 1.54 | 0.02 | 0.20 | 1.96 | 0.97 |  |
| 42 | Ergosterol peroxide | 429.3 | 85.1 | 13.49 | 9.41E-06 | 1.86E-05 | 1.63 | 0.02 | 0.19 | 0.51 | -0.98 |  |
| 43 | Estriol | 289.2 | 253.2 | 5.78 | 9.74E-06 | 3.18E-06 | 1.67 | 0.02 | 0.22 | 3.06 | 1.62 |  |
| 44 | Ethyl caproate | 145.1 | 57.1 | 0.74 | 2.03E-04 | 4.16E-04 | 1.74 | 0.00 | 0.11 | 0.49 | -1.04 |  |
| 45 | Ethyl trans-p-methoxycinnamate | 207.1 | 133.1 | 4.97 | 1.50E-04 | 1.07E-04 | 1.53 | 0.05 | 0.30 | 1.40 | 0.49 |  |
| 46 | Exemestane | 297.2 | 121.1 | 11.61 | 2.72E-04 | 1.72E-04 | 1.75 | 0.01 | 0.17 | 1.58 | 0.66 |  |
| 47 | Ferulic acid | 195.1 | 117 | 4.86 | 5.19E-04 | 2.79E-04 | 1.76 | 0.00 | 0.10 | 1.86 | 0.90 |  |
| 48 | Ganoderal A | 437.3 | 419.3 | 8.09 | 1.79E-05 | 6.88E-06 | 1.83 | 0.00 | 0.07 | 2.60 | 1.38 |  |
| 49 | Ganoderol A | 439.4 | 421.3 | 11.31 | 4.57E-05 | 1.73E-05 | 1.66 | 0.00 | 0.11 | 2.64 | 1.40 |  |
| 50 | Garbanzol | 273.1 | 107 | 9.90 | 6.42E-05 | 4.56E-05 | 1.54 | 0.04 | 0.27 | 1.41 | 0.49 |  |
| 51 | Glucosamine | 180.1 | 162.1 | 2.60 | 8.11E-04 | 5.96E-04 | 1.60 | 0.02 | 0.22 | 1.36 | 0.44 |  |
| 52 | Grandifloric acid | 319.2 | 255.2 | 6.48 | 2.74E-05 | 1.33E-05 | 1.46 | 0.03 | 0.22 | 2.05 | 1.04 |  |
| 53 | Homopterocarpin | 285.1 | 107 | 9.07 | 7.71E-05 | 2.79E-05 | 1.52 | 0.01 | 0.14 | 2.76 | 1.47 |  |
| 54 | Indole-3-acetic acid | 176 | 130 | 7.14 | 2.00E-04 | 1.15E-04 | 1.61 | 0.01 | 0.16 | 1.74 | 0.80 |  |
| 55 | Indolin-2-one | 134.1 | 77 | 5.67 | 4.15E-05 | 2.72E-05 | 1.61 | 0.03 | 0.25 | 1.53 | 0.61 |  |
| 56 | Isoquinoline | 130.1 | 103.1 | 6.13 | 2.76E-04 | 2.15E-04 | 1.58 | 0.04 | 0.28 | 1.28 | 0.36 |  |
| 57 | Kaempferol 3-O-beta-sophoroside | 611.2 | 287.1 | 6.83 | 2.67E-04 | 3.31E-04 | 1.59 | 0.04 | 0.26 | 0.81 | -0.31 |  |
| 58 | Ketopantoic acid | 147.1 | 73.1 | 0.70 | 2.98E-04 | 4.31E-04 | 1.58 | 0.02 | 0.22 | 0.69 | -0.53 |  |
| 59 | Kojibiose | 325.1 | 85 | 0.72 | 3.51E-03 | 6.69E-03 | 1.63 | 0.02 | 0.21 | 0.53 | -0.93 |  |
| 60 | L-arginine | 175.1 | 70.05 | 0.60 | 7.34E-03 | 2.36E-02 | 1.45 | 0.00 | 0.11 | 0.31 | -1.69 |  |
| 61 | L-asparagine | 133.1 | 74 | 0.64 | 2.70E-04 | 4.31E-04 | 1.68 | 0.02 | 0.21 | 0.63 | -0.67 |  |
| 62 | L-Aspartic acid | 134 | 74 | 0.63 | 5.11E-05 | 7.90E-05 | 1.67 | 0.02 | 0.20 | 0.65 | -0.63 |  |
| 63 | L-Glutamic acid | 148.1 | 84 | 0.66 | 3.62E-03 | 5.51E-03 | 1.67 | 0.03 | 0.23 | 0.66 | -0.61 |  |
| 64 | L-lysine | 147.1 | 84.067 | 0.64 | 5.09E-03 | 1.16E-02 | 1.85 | 0.00 | 0.04 | 0.44 | -1.18 |  |
| 65 | L-phenylalanine | 166.1 | 120.1 | 2.82 | 2.53E-02 | 8.13E-03 | 1.21 | 0.01 | 0.18 | 3.11 | 1.64 |  |
| 66 | Leukotriene A4 | 319.2 | 301.2 | 5.57 | 3.86E-05 | 1.37E-05 | 1.69 | 0.02 | 0.20 | 2.81 | 1.49 |  |
| 67 | Linolenic acid | 277.2 | 59 | 12.17 | 5.65E-06 | 1.75E-05 | 1.65 | 0.01 | 0.17 | 0.32 | -1.63 |  |
| 68 | Liquiritin | 417 | 254.9 | 6.00 | 1.82E-05 | 9.51E-06 | 1.61 | 0.02 | 0.21 | 1.91 | 0.93 |  |
| 69 | Malvidin-3-O-galactoside | 494.1 | 331.1 | 7.75 | 7.43E-06 | 2.54E-06 | 1.70 | 0.01 | 0.15 | 2.92 | 1.55 |  |
| 70 | Mesaconitine | 632.3 | 105 | 12.92 | 1.40E-06 | 1.10E-05 | 1.48 | 0.00 | 0.08 | 0.13 | -2.98 |  |
| 71 | Methyl (indol-3-yl)acetate | 190.05 | 130.05 | 9.48 | 4.57E-05 | 1.22E-05 | 1.47 | 0.02 | 0.20 | 3.75 | 1.91 |  |
| 72 | N-((-)-jasmonoyl)-S-isoleucine | 322 | 130 | 10.41 | 1.35E-03 | 9.06E-04 | 1.65 | 0.01 | 0.17 | 1.49 | 0.57 |  |
| 73 | N-Acetyl-D-glucosamine 6-phosphate | 302.1 | 99 | 5.50 | 3.83E-05 | 2.37E-05 | 1.62 | 0.02 | 0.20 | 1.62 | 0.69 |  |
| 74 | N-Acetyl-L-glutamate 5-semialdehyde | 174.1 | 86.1 | 1.51 | 1.12E-04 | 2.42E-04 | 1.60 | 0.02 | 0.19 | 0.46 | -1.12 |  |
| 75 | N-Feruloyl putrescine | 265.2 | 72.1 | 4.33 | 2.40E-04 | 1.70E-04 | 1.53 | 0.04 | 0.27 | 1.41 | 0.50 |  |
| 76 | N,n-dimethylsphingosine | 326.3 | 296.3 | 8.38 | 2.48E-05 | 4.49E-05 | 1.63 | 0.03 | 0.25 | 0.55 | -0.86 |  |
| 77 | N1-(alpha-D-ribosyl)-5,6-dimethyl-benzimidazole | 279.1 | 147.1 | 11.88 | 1.26E-04 | 3.16E-04 | 1.73 | 0.02 | 0.21 | 0.40 | -1.33 |  |
| 78 | N1-Methyl-4-pyridone-3-carboxamide | 153.1 | 136 | 2.66 | 9.23E-05 | 5.64E-05 | 1.80 | 0.00 | 0.07 | 1.64 | 0.71 |  |
| 79 | Napellonine | 358.2 | 340.2 | 8.48 | 1.69E-03 | 4.59E-04 | 1.74 | 0.01 | 0.12 | 3.68 | 1.88 |  |
| 80 | Naringenin | 273.3 | 152.8 | 8.86 | 1.51E-05 | 4.84E-06 | 1.66 | 0.02 | 0.20 | 3.13 | 1.65 |  |
| 81 | Nicotinurate | 181.1 | 135.1 | 3.27 | 7.06E-05 | 5.11E-05 | 1.67 | 0.02 | 0.21 | 1.38 | 0.47 |  |
| 82 | Norlichexanthone | 259.1 | 241.1 | 4.48 | 2.41E-05 | 8.61E-06 | 1.74 | 0.01 | 0.12 | 2.80 | 1.48 |  |
| 83 | Octadecanamide | 284.3 | 43.1 | 13.02 | 3.17E-04 | 4.09E-04 | 1.56 | 0.03 | 0.26 | 0.78 | -0.36 |  |
| 84 | Phenethylamine | 122.1 | 105.1 | 2.82 | 1.11E-04 | 5.43E-05 | 1.72 | 0.00 | 0.10 | 2.05 | 1.04 |  |
| 85 | Phloretic acid | 167.1 | 121.07 | 2.84 | 2.31E-03 | 1.25E-03 | 1.70 | 0.00 | 0.10 | 1.84 | 0.88 |  |
| 86 | Phosphoric acid | 99 | 81 | 0.65 | 4.62E-04 | 7.07E-04 | 1.59 | 0.04 | 0.28 | 0.65 | -0.61 |  |
| 87 | Phosphorylcholine | 184.1 | 86.1 | 0.67 | 3.69E-04 | 6.48E-04 | 1.76 | 0.01 | 0.14 | 0.57 | -0.81 |  |
| 88 | Pilocarpine | 209.1 | 95.1 | 5.04 | 1.14E-04 | 6.21E-05 | 1.78 | 0.00 | 0.11 | 1.83 | 0.88 |  |
| 89 | Pogostone | 225.1 | 71.1 | 4.95 | 1.75E-05 | 8.61E-06 | 1.42 | 0.04 | 0.29 | 2.03 | 1.02 |  |
| 90 | L-proline | 116.1 | 70.1 | 0.73 | 2.99E-02 | 3.20E-02 | 1.81 | 0.00 | 0.08 | 0.94 | -0.10 |  |
| 91 | Prostaglandin I2 | 353.2 | 317.2 | 9.39 | 1.47E-04 | 3.03E-04 | 1.76 | 0.01 | 0.14 | 0.48 | -1.05 |  |
| 92 | Protopine | 354.1 | 188.1 | 11.45 | 1.82E-05 | 4.53E-05 | 1.44 | 0.03 | 0.25 | 0.40 | -1.31 |  |
| 93 | Pseudotropine | 142.1 | 124.1 | 0.81 | 1.28E-02 | 1.61E-02 | 1.55 | 0.04 | 0.28 | 0.80 | -0.33 |  |
| 94 | Pyridoxine | 170.1 | 134.1 | 1.55 | 2.36E-04 | 1.46E-04 | 1.63 | 0.03 | 0.22 | 1.62 | 0.70 |  |
| 95 | Rutin | 611.2 | 303.1 | 5.94 | 3.21E-05 | 2.57E-06 | 1.66 | 0.00 | 0.08 | 12.53 | 3.65 |  |
| 96 | Sinapyl alcohol | 193.1 | 161.1 | 4.43 | 5.46E-06 | 7.30E-06 | 1.56 | 0.04 | 0.27 | 0.75 | -0.42 |  |
| 97 | Swertiaperennin | 289.1 | 165 | 5.12 | 1.25E-05 | 5.36E-06 | 1.68 | 0.02 | 0.20 | 2.34 | 1.23 |  |
| 98 | Talatisamine | 422.3 | 404.3 | 4.66 | 2.31E-05 | 1.24E-05 | 1.77 | 0.01 | 0.12 | 1.86 | 0.90 |  |
| 99 | Thromboxane A2 | 353.2 | 71.1 | 11.43 | 4.92E-05 | 1.18E-04 | 1.75 | 0.00 | 0.11 | 0.42 | -1.26 |  |
| 100 | Trans-4-Hydroxy-L-proline | 132.1 | 86.05 | 0.65 | 1.60E-04 | 3.42E-04 | 1.77 | 0.01 | 0.17 | 0.47 | -1.09 |  |
| 101 | Undecanolactone | 185.2 | 43.1 | 0.57 | 2.87E-04 | 3.71E-04 | 1.71 | 0.01 | 0.18 | 0.77 | -0.37 |  |
| 102 | Uridine 5'-diphospho-D-glucose | 565 | 323 | 0.71 | 1.64E-04 | 3.45E-04 | 1.54 | 0.04 | 0.27 | 0.48 | -1.07 |  |
| 103 | Urocanic acid | 139.1 | 93 | 4.21 | 9.71E-05 | 6.44E-05 | 1.85 | 0.01 | 0.14 | 1.51 | 0.59 |  |
| 104 | Vasicine | 189.1 | 171.1 | 3.76 | 6.62E-04 | 4.47E-04 | 1.47 | 0.04 | 0.27 | 1.48 | 0.57 |  |

| Differential metabolites of nano-selenium (5 mg/L) and control in pepper root | | | | | | | | | | | |
| --- | --- | --- | --- | --- | --- | --- | --- | --- | --- | --- | --- |
| Number | compound name | Q1 | Q3 | RT | MEAN T1 | MEAN CK | VIP | P-VALUE | Q-VALUE | FOLD CHANGE | LOG_  FOLDCHANGE |
| 1 | (-)-Epicatechin gallate | 443.1 | 123 | 6.20 | 1.13E-03 | 8.86E-04 | 1.61 | 0.035 | 0.30 | 1.28 | 0.35 |
| 2 | (±)-Jasmonic acid | 209 | 59 | 8.94 | 4.78E-04 | 5.81E-04 | 1.78 | 0.010 | 0.18 | 0.82 | -0.28 |
| 3 | (S)-2-Aceto-2-hydroxybutanoate | 147.1 | 57 | 0.66 | 5.17E-05 | 8.55E-05 | 1.88 | 0.001 | 0.16 | 0.60 | -0.73 |
| 4 | (S)-2,3,4,5-tetrahydropyridine-2-carboxylate | 128.1 | 82.1 | 1.74 | 1.16E-04 | 6.62E-05 | 1.71 | 0.010 | 0.18 | 1.76 | 0.82 |
| 5 | 1-Naphthylacetic acid | 187.1 | 141.1 | 10.55 | 2.24E-05 | 1.49E-05 | 1.69 | 0.038 | 0.31 | 1.51 | 0.59 |
| 6 | 1H-Indole-2,3-dione | 148 | 120 | 2.79 | 2.87E-05 | 1.77E-05 | 1.59 | 0.033 | 0.30 | 1.62 | 0.70 |
| 7 | 3-butylidenephthalide | 189.1 | 79.1 | 8.74 | 1.21E-05 | 8.05E-06 | 1.66 | 0.023 | 0.27 | 1.50 | 0.58 |
| 8 | 3-Carbamyl-1-methylpyridinium (1-Methylnicotinamide) | 137.1 | 94.1 | 6.36 | 4.59E-04 | 3.36E-04 | 1.66 | 0.033 | 0.30 | 1.37 | 0.45 |
| 9 | 3-Hydroxy-4-methoxycinnamic acid | 195.1 | 149.1 | 4.87 | 1.06E-04 | 6.88E-05 | 1.60 | 0.034 | 0.30 | 1.54 | 0.63 |
| 10 | 3-Hydroxybenzoic acid | 139 | 121 | 8.39 | 1.53E-05 | 8.46E-06 | 1.75 | 0.022 | 0.26 | 1.81 | 0.86 |
| 11 | 3-Methoxy-4,5-methylenedioxycinnamaldehyde | 207.1 | 189.1 | 5.04 | 8.88E-05 | 6.13E-05 | 1.59 | 0.029 | 0.29 | 1.45 | 0.53 |
| 12 | 4-methylumbelliferone | 177.1 | 149.1 | 7.26 | 9.35E-06 | 1.74E-05 | 1.59 | 0.033 | 0.30 | 0.54 | -0.89 |
| 13 | 6-Amino-2-oxohexanoate | 146.1 | 72.1 | 0.77 | 4.13E-03 | 8.08E-03 | 1.88 | 0.001 | 0.16 | 0.51 | -0.97 |
| 14 | 6,7,4'-trihydroxyisoflavone | 271.1 | 105 | 10.76 | 2.06E-05 | 6.81E-06 | 1.62 | 0.007 | 0.17 | 3.02 | 1.59 |
| 15 | 8(r)-hpete | 337.2 | 149.1 | 11.58 | 3.18E-04 | 4.83E-04 | 1.56 | 0.042 | 0.32 | 0.66 | -0.60 |
| 16 | Allysine(6-oxo dl-norleucine) | 146.1 | 100.1 | 0.77 | 2.66E-02 | 4.87E-02 | 1.84 | 0.002 | 0.16 | 0.55 | -0.87 |
| 17 | Apiin | 565.5 | 432.9 | 6.46 | 7.73E-05 | 9.03E-04 | 1.92 | 0.008 | 0.17 | 0.09 | -3.55 |
| 18 | Benzaldehyde | 107 | 79 | 2.82 | 3.15E-04 | 1.65E-04 | 1.77 | 0.004 | 0.17 | 1.91 | 0.93 |
| 19 | Benzoylagmatine | 235.2 | 105 | 10.56 | 2.42E-05 | 6.54E-05 | 1.59 | 0.023 | 0.26 | 0.37 | -1.43 |
| 20 | Beta-Nicotinamide mononucleotide | 335.1 | 123.1 | 0.73 | 3.18E-04 | 6.91E-04 | 1.81 | 0.002 | 0.16 | 0.46 | -1.12 |
| 21 | Bovinic acid | 303.2 | 183 | 6.86 | 3.01E-05 | 1.58E-05 | 1.87 | 0.003 | 0.17 | 1.90 | 0.92 |
| 22 | Chrysin dimethylether | 283.1 | 239.1 | 11.39 | 1.83E-06 | 1.21E-05 | 1.42 | 0.025 | 0.27 | 0.15 | -2.72 |
| 23 | Cis-Aconitic acid | 175 | 69 | 1.46 | 7.51E-03 | 1.19E-02 | 1.77 | 0.005 | 0.17 | 0.63 | -0.67 |
| 24 | Colneleate | 295.2 | 123.1 | 11.97 | 2.70E-03 | 2.02E-03 | 1.60 | 0.042 | 0.32 | 1.33 | 0.42 |
| 25 | D-glutamine | 147.1 | 84 | 0.66 | 6.29E-03 | 1.16E-02 | 1.91 | 0.000 | 0.15 | 0.54 | -0.88 |
| 26 | D-maltose | 341.1 | 59 | 0.76 | 1.63E-02 | 3.26E-02 | 1.86 | 0.002 | 0.16 | 0.50 | -1.00 |
| 27 | Daphnoretin | 353.1 | 163 | 11.45 | 2.69E-04 | 5.26E-04 | 1.59 | 0.047 | 0.33 | 0.51 | -0.96 |
| 28 | Deethylatrazine | 188.1 | 146 | 3.81 | 1.23E-02 | 7.96E-03 | 1.79 | 0.004 | 0.17 | 1.54 | 0.62 |
| 29 | Denudatine | 344.3 | 326.2 | 1.59 | 1.78E-03 | 1.11E-03 | 1.75 | 0.022 | 0.26 | 1.60 | 0.68 |
| 30 | Dg(16:0/16:0/0:0) | 591.5 | 313.3 | 13.42 | 1.23E-04 | 4.92E-05 | 1.71 | 0.003 | 0.17 | 2.51 | 1.33 |
| 31 | Diallyl disulfide | 147 | 41 | 0.68 | 4.19E-04 | 6.22E-04 | 1.72 | 0.035 | 0.30 | 0.67 | -0.57 |
| 32 | Diphyllin | 381.1 | 335.1 | 10.09 | 4.15E-06 | 2.09E-06 | 1.67 | 0.033 | 0.30 | 1.98 | 0.99 |
| 33 | Dump | 309 | 113 | 0.76 | 3.33E-05 | 1.43E-05 | 1.70 | 0.007 | 0.17 | 2.32 | 1.22 |
| 34 | Ent-16beta,17-dihydroxy-9(11)-kauren-19-oic acid | 335.2 | 271.2 | 7.15 | 1.67E-05 | 7.36E-06 | 1.79 | 0.007 | 0.17 | 2.27 | 1.18 |
| 35 | Ethyl acrylate | 101.1 | 55 | 1.74 | 1.10E-04 | 1.82E-04 | 1.83 | 0.009 | 0.17 | 0.60 | -0.73 |
| 36 | Ethyl caproate | 145.1 | 57.1 | 0.74 | 2.08E-04 | 4.16E-04 | 1.78 | 0.005 | 0.17 | 0.50 | -1.00 |
| 37 | Forskolin | 409.2 | 59 | 5.45 | 9.77E-06 | 4.55E-06 | 1.59 | 0.034 | 0.30 | 2.15 | 1.10 |
| 38 | Galactose 1-phosphate | 259 | 79 | 0.65 | 1.32E-04 | 4.01E-04 | 1.77 | 0.041 | 0.32 | 0.33 | -1.61 |
| 39 | Ganoderic acid S | 453.3 | 391.3 | 9.21 | 5.90E-05 | 1.27E-04 | 1.78 | 0.010 | 0.18 | 0.46 | -1.11 |
| 40 | Ganoderol B | 441.4 | 405.4 | 11.21 | 2.50E-05 | 8.47E-06 | 1.79 | 0.022 | 0.26 | 2.95 | 1.56 |
| 41 | Garbanzol | 273.1 | 107 | 9.90 | 6.76E-05 | 4.56E-05 | 1.66 | 0.027 | 0.28 | 1.48 | 0.57 |
| 42 | Ginsenoside F1 | 639.4 | 603.4 | 9.11 | 7.95E-06 | 3.95E-06 | 1.44 | 0.036 | 0.31 | 2.01 | 1.01 |
| 43 | Ginsenoside rf | 801.5 | 765.5 | 9.01 | 7.57E-06 | 1.46E-06 | 1.40 | 0.027 | 0.28 | 5.17 | 2.37 |
| 44 | Glucosamine | 180.1 | 162.1 | 2.60 | 9.64E-04 | 5.96E-04 | 1.82 | 0.003 | 0.17 | 1.62 | 0.69 |
| 45 | Guaiacol | 125.1 | 65 | 5.62 | 2.19E-04 | 1.79E-04 | 1.63 | 0.035 | 0.30 | 1.22 | 0.29 |
| 46 | Guanosine 3',5'-cyclic monophosphate | 346.1 | 152.1 | 2.26 | 7.67E-05 | 4.29E-05 | 1.62 | 0.024 | 0.27 | 1.79 | 0.84 |
| 47 | Hordenine | 166.1 | 121.1 | 3.04 | 1.60E-03 | 1.07E-03 | 1.74 | 0.011 | 0.18 | 1.50 | 0.58 |
| 48 | Indole | 118.1 | 91.1 | 3.82 | 1.14E-02 | 8.78E-03 | 1.83 | 0.004 | 0.17 | 1.30 | 0.38 |
| 49 | Indole-3-acetic acid | 176 | 130 | 7.14 | 2.01E-04 | 1.15E-04 | 1.62 | 0.028 | 0.28 | 1.75 | 0.80 |
| 50 | Kaempferol 3-O-beta-sophoroside | 611.2 | 287.1 | 6.83 | 2.35E-04 | 3.31E-04 | 1.55 | 0.039 | 0.31 | 0.71 | -0.50 |
| 51 | Karanjin | 293.1 | 105 | 11.67 | 7.82E-04 | 5.90E-04 | 1.78 | 0.007 | 0.17 | 1.32 | 0.40 |
| 52 | Ketopantoic acid | 147.1 | 73.1 | 0.70 | 2.34E-04 | 4.31E-04 | 1.77 | 0.005 | 0.17 | 0.54 | -0.88 |
| 53 | Kojibiose | 325.1 | 85 | 0.72 | 3.52E-03 | 6.69E-03 | 1.71 | 0.020 | 0.25 | 0.53 | -0.92 |
| 54 | L-arginine | 175.1 | 70.05 | 0.60 | 4.85E-03 | 2.36E-02 | 1.68 | 0.002 | 0.16 | 0.21 | -2.29 |
| 55 | L-asparagine | 133.1 | 74 | 0.64 | 2.32E-04 | 4.31E-04 | 1.64 | 0.024 | 0.27 | 0.54 | -0.90 |
| 56 | L-citruline | 176.1 | 70.1 | 0.66 | 3.09E-04 | 8.93E-04 | 1.64 | 0.016 | 0.23 | 0.35 | -1.53 |
| 57 | L-homocitrulline | 190.1 | 101.1 | 0.72 | 4.15E-05 | 2.66E-05 | 1.80 | 0.006 | 0.17 | 1.56 | 0.64 |
| 58 | L-Homoglutamic acid | 162.1 | 98.1 | 0.73 | 1.12E-04 | 8.30E-05 | 1.60 | 0.037 | 0.31 | 1.35 | 0.43 |
| 59 | L-lysine | 147.1 | 84.06667 | 0.64 | 6.29E-03 | 1.16E-02 | 1.91 | 0.000 | 0.15 | 0.54 | -0.88 |
| 60 | L-phenylalanine | 166.1 | 120.1 | 2.82 | 2.65E-02 | 8.13E-03 | 1.27 | 0.040 | 0.31 | 3.26 | 1.71 |
| 61 | L-Pipecolic acid | 130.1 | 84.1 | 0.69 | 6.99E-03 | 8.88E-03 | 1.90 | 0.010 | 0.18 | 0.79 | -0.34 |
| 62 | Pipecolic acid | 130.1 | 84.1 | 1.47 | 2.01E-03 | 2.72E-03 | 1.62 | 0.043 | 0.32 | 0.74 | -0.44 |
| 63 | Linolenic acid | 277.2 | 59 | 12.17 | 4.07E-06 | 1.75E-05 | 1.84 | 0.003 | 0.17 | 0.23 | -2.10 |
| 64 | Lipiferolide | 307.2 | 247.1 | 5.36 | 2.06E-05 | 1.06E-05 | 1.58 | 0.038 | 0.31 | 1.95 | 0.96 |
| 65 | Maltotriose | 527.2 | 365.1 | 1.23 | 5.00E-03 | 7.30E-03 | 1.66 | 0.040 | 0.31 | 0.68 | -0.55 |
| 66 | N-(p-Hydroxyphenethyl)actinidine | 269.2 | 121.1 | 9.87 | 6.47E-06 | 9.58E-06 | 1.58 | 0.032 | 0.30 | 0.67 | -0.57 |
| 67 | N-Acetyl-L-glutamate 5-semialdehyde | 174.1 | 86.1 | 1.51 | 1.10E-04 | 2.42E-04 | 1.77 | 0.005 | 0.17 | 0.46 | -1.13 |
| 68 | N,n-dimethylsphingosine | 326.3 | 296.3 | 8.38 | 2.32E-05 | 4.49E-05 | 1.74 | 0.024 | 0.27 | 0.52 | -0.95 |
| 69 | Napellonine | 358.2 | 340.2 | 8.48 | 1.28E-03 | 4.59E-04 | 1.73 | 0.008 | 0.17 | 2.79 | 1.48 |
| 70 | Nicotinurate | 181.1 | 135.1 | 3.27 | 5.88E-05 | 5.11E-05 | 1.82 | 0.025 | 0.27 | 1.15 | 0.20 |
| 71 | Norepinephrine | 170.1 | 152.1 | 1.85 | 8.59E-06 | 6.25E-06 | 1.63 | 0.031 | 0.29 | 1.37 | 0.46 |
| 72 | Octadecanamide | 284.3 | 43.1 | 13.02 | 3.16E-04 | 4.09E-04 | 1.64 | 0.030 | 0.29 | 0.77 | -0.37 |
| 73 | P-Cresol | 109.1 | 94 | 7.28 | 1.83E-05 | 1.04E-05 | 1.60 | 0.024 | 0.27 | 1.76 | 0.81 |
| 74 | Petasitenine | 382.2 | 364.2 | 4.68 | 2.63E-05 | 1.13E-05 | 1.64 | 0.041 | 0.32 | 2.33 | 1.22 |
| 75 | Phenethylamine | 122.1 | 105.1 | 2.82 | 9.68E-05 | 5.43E-05 | 1.70 | 0.012 | 0.20 | 1.78 | 0.83 |
| 76 | Phloretic acid | 167.1 | 121.0667 | 2.84 | 2.48E-03 | 1.25E-03 | 1.75 | 0.008 | 0.17 | 1.98 | 0.99 |
| 77 | Phosphorylcholine | 184.1 | 86.1 | 0.67 | 3.54E-04 | 6.48E-04 | 1.78 | 0.012 | 0.19 | 0.55 | -0.87 |
| 78 | Piperidine | 86.1 | 69.1 | 2.25 | 7.82E-06 | 2.15E-05 | 1.83 | 0.001 | 0.16 | 0.36 | -1.46 |
| 79 | Piperitenone | 151.1 | 39 | 9.92 | 5.47E-06 | 2.80E-06 | 1.67 | 0.017 | 0.23 | 1.96 | 0.97 |
| 80 | Porson | 387.2 | 313.1 | 7.45 | 2.80E-06 | 8.19E-06 | 1.60 | 0.043 | 0.32 | 0.34 | -1.55 |
| 81 | Pseudotropine | 142.1 | 124.1 | 0.81 | 1.05E-02 | 1.61E-02 | 1.83 | 0.004 | 0.17 | 0.65 | -0.61 |
| 82 | Pterosin D | 249.1 | 231.1 | 3.29 | 3.99E-05 | 1.74E-05 | 1.50 | 0.020 | 0.25 | 2.29 | 1.20 |
| 83 | Pyrrolidonecarboxylic acid | 130 | 84 | 0.67 | 6.99E-03 | 8.88E-03 | 1.90 | 0.010 | 0.18 | 0.79 | -0.34 |
| 84 | Simvastatin | 419.3 | 199.1 | 8.07 | 6.97E-06 | 3.25E-06 | 1.84 | 0.003 | 0.17 | 2.15 | 1.10 |
| 85 | Sinapyl alcohol | 193.1 | 161.1 | 4.43 | 1.00E-05 | 7.30E-06 | 1.78 | 0.008 | 0.17 | 1.37 | 0.45 |
| 86 | Stachyose | 689.2 | 527.2 | 0.73 | 3.96E-04 | 1.08E-03 | 1.71 | 0.028 | 0.28 | 0.37 | -1.44 |
| 87 | Tabernanthine | 311.2 | 138.1 | 3.79 | 3.21E-06 | 1.18E-06 | 1.59 | 0.047 | 0.33 | 2.71 | 1.44 |
| 88 | Taxiphyllin | 312.1 | 132 | 13.76 | 3.12E-06 | 4.22E-06 | 1.66 | 0.017 | 0.24 | 0.74 | -0.44 |
| 89 | Tectochrysin | 269.1 | 251.1 | 9.88 | 5.09E-06 | 3.07E-06 | 1.60 | 0.038 | 0.31 | 1.66 | 0.73 |
| 90 | Thromboxane A2 | 353.2 | 71.1 | 11.43 | 5.15E-05 | 1.18E-04 | 1.62 | 0.019 | 0.24 | 0.44 | -1.20 |
| 91 | Trans-4-Hydroxy-L-proline | 132.1 | 86.05 | 0.65 | 1.14E-04 | 3.42E-04 | 1.82 | 0.007 | 0.17 | 0.33 | -1.58 |
| 92 | Trans-caffeic acid | 181 | 121 | 3.31 | 5.66E-05 | 2.30E-05 | 1.76 | 0.007 | 0.17 | 2.47 | 1.30 |
| 93 | Tryptophan | 205.1 | 188.1 | 3.77 | 3.95E-04 | 1.49E-04 | 1.00 | 0.036 | 0.30 | 2.65 | 1.41 |
| 94 | Undecanolactone | 185.2 | 43.1 | 0.57 | 2.72E-04 | 3.71E-04 | 1.75 | 0.015 | 0.22 | 0.73 | -0.45 |
| 95 | Uridine 5'-diphospho-D-glucose | 565 | 323 | 0.71 | 1.89E-04 | 3.45E-04 | 1.68 | 0.030 | 0.29 | 0.55 | -0.87 |
| 96 | Vanillic acid | 169 | 65 | 3.78 | 1.33E-03 | 9.85E-04 | 1.73 | 0.014 | 0.21 | 1.35 | 0.43 |
| 97 | Vestitol | 273.1 | 123 | 6.51 | 5.68E-06 | 3.81E-05 | 1.74 | 0.006 | 0.17 | 0.15 | -2.75 |
| 98 | Yohimbine | 353.2 | 335.2 | 12.13 | 1.36E-05 | 1.75E-05 | 1.61 | 0.043 | 0.32 | 0.77 | -0.37 |

| Differential metabolites of nano-selenium (20 mg/L) and control in pepper root | | | | | | | | | | | | |
| --- | --- | --- | --- | --- | --- | --- | --- | --- | --- | --- | --- | --- |
| Number | Compound | Q1 | Q3 | RT | Mean T1 | Mean CK | VIP | P-value | Q-value | Fold Change | LOG_Fold Change |  |
| 1 | (S)-2-Aceto-2-hydroxybutanoate | 147.1 | 57 | 0.66 | 1.37E-04 | 8.55E-05 | 1.83 | 0.001 | 0.051 | 1.60 | 0.67 |  |
| 2 | 16(r)-hete | 303.2 | 91.1 | 6.87 | 3.15E-05 | 6.06E-05 | 1.69 | 0.019 | 0.19 | 0.52 | -0.94 |  |
| 3 | 2-hydroxyethanesulfonate | 127 | 109 | 0.76 | 5.37E-05 | 8.73E-05 | 1.59 | 0.034 | 0.24 | 0.61 | -0.70 |  |
| 4 | 2-Isopropyl-3-oxosuccinate | 175.1 | 129.1 | 0.80 | 1.02E-04 | 1.38E-04 | 1.58 | 0.033 | 0.24 | 0.74 | -0.44 |  |
| 5 | 2-pentylfuran | 139.1 | 55.1 | 1.11 | 7.19E-03 | 1.02E-02 | 1.78 | 0.003 | 0.10 | 0.71 | -0.50 |  |
| 6 | 2,4-dimethylphenol | 123.1 | 65 | 10.56 | 1.05E-05 | 2.19E-05 | 1.60 | 0.040 | 0.26 | 0.48 | -1.07 |  |
| 7 | 3,4-dihydroxyphenylacetaldehyde | 153.1 | 135 | 4.05 | 6.04E-06 | 8.90E-06 | 1.59 | 0.023 | 0.21 | 0.68 | -0.56 |  |
| 8 | 4-hydroxybenzylamine | 124.1 | 107 | 2.30 | 4.65E-06 | 2.20E-06 | 1.59 | 0.032 | 0.24 | 2.11 | 1.08 |  |
| 9 | 4-Methyl-5-thiazoleethanol | 144 | 113 | 2.66 | 3.43E-04 | 2.38E-04 | 1.53 | 0.047 | 0.28 | 1.44 | 0.53 |  |
| 10 | 5-oxoproline | 152 | 69 | 0.56 | 1.03E-03 | 1.41E-03 | 1.59 | 0.036 | 0.25 | 0.73 | -0.45 |  |
| 11 | 5,6-dhet | 339.3 | 303.2 | 13.03 | 8.96E-05 | 1.46E-04 | 1.61 | 0.048 | 0.28 | 0.62 | -0.70 |  |
| 12 | 5,6-dihydroxyindole | 150.1 | 78 | 6.68 | 7.05E-06 | 1.42E-05 | 1.60 | 0.020 | 0.20 | 0.50 | -1.01 |  |
| 13 | 5,7-dihydroxyisoflavone | 242.1 | 224.1 | 14.24 | 1.40E-06 | 9.21E-07 | 1.54 | 0.038 | 0.25 | 1.52 | 0.61 |  |
| 14 | 6-gingerol | 293.2 | 57 | 11.39 | 7.71E-05 | 2.92E-05 | 1.83 | 0.002 | 0.08 | 2.64 | 1.40 |  |
| 15 | 7-hydroxyflavone | 239.1 | 137 | 0.56 | 6.84E-05 | 1.20E-04 | 1.66 | 0.028 | 0.23 | 0.57 | -0.81 |  |
| 16 | 9-oxoode | 295.2 | 277.2 | 11.98 | 2.46E-03 | 1.22E-03 | 1.77 | 0.002 | 0.08 | 2.02 | 1.02 |  |
| 17 | Alpha-caryophyllene | 205.2 | 81.1 | 11.58 | 1.50E-05 | 4.23E-05 | 1.70 | 0.013 | 0.17 | 0.36 | -1.49 |  |
| 18 | Antirhine | 297.2 | 279.2 | 11.61 | 1.06E-05 | 8.86E-05 | 1.45 | 0.040 | 0.26 | 0.12 | -3.06 |  |
| 19 | Apiin | 565.5 | 432.9 | 6.46 | 3.22E-04 | 9.03E-04 | 1.73 | 0.004 | 0.10 | 0.36 | -1.49 |  |
| 20 | Benzaldehyde | 107 | 79 | 2.82 | 2.64E-04 | 1.65E-04 | 1.60 | 0.017 | 0.19 | 1.60 | 0.68 |  |
| 21 | Benzoylagmatine | 235.2 | 105 | 10.56 | 2.07E-05 | 6.54E-05 | 1.79 | 0.003 | 0.096 | 0.32 | -1.66 |  |
| 22 | Beta-Nicotinamide mononucleotide | 335.1 | 123.1 | 0.73 | 2.97E-04 | 6.91E-04 | 1.80 | 0.001 | 0.067 | 0.43 | -1.22 |  |
| 23 | Canthaxanthin | 565.4 | 203.1 | 12.82 | 6.95E-06 | 1.16E-05 | 1.74 | 0.007 | 0.14 | 0.60 | -0.74 |  |
| 24 | Chrysin dimethylether | 283.1 | 239.1 | 11.39 | 2.34E-06 | 1.21E-05 | 1.31 | 0.036 | 0.25 | 0.19 | -2.37 |  |
| 25 | Cinchonine | 295.2 | 79.1 | 11.99 | 1.81E-02 | 8.08E-03 | 1.83 | 0.000 | 0.043 | 2.24 | 1.16 |  |
| 26 | Cinnamyl alcohol | 135.1 | 117.1 | 10.56 | 1.52E-05 | 2.59E-05 | 1.63 | 0.039 | 0.26 | 0.59 | -0.77 |  |
| 27 | Cis-Aconitic acid | 175 | 69 | 1.46 | 8.01E-03 | 1.19E-02 | 1.80 | 0.001 | 0.068 | 0.67 | -0.58 |  |
| 28 | Colneleate | 295.2 | 123.1 | 11.97 | 2.96E-03 | 2.02E-03 | 1.61 | 0.036 | 0.249 | 1.46 | 0.55 |  |
| 29 | Crotonoside | 284.1 | 152.1 | 2.40 | 5.17E-03 | 7.26E-03 | 1.77 | 0.003 | 0.095 | 0.71 | -0.49 |  |
| 30 | Curzerene | 217.2 | 55.1 | 2.55 | 5.38E-05 | 7.33E-05 | 1.57 | 0.031 | 0.24 | 0.73 | -0.45 |  |
| 31 | Cynaroside | 449 | 286.9 | 6.10 | 3.35E-05 | 6.29E-05 | 1.52 | 0.042 | 0.26 | 0.53 | -0.91 |  |
| 32 | Cytidine | 244.1 | 112.1 | 0.70 | 9.15E-05 | 1.09E-04 | 1.57 | 0.043 | 0.27 | 0.84 | -0.25 |  |
| 33 | D-Fructose 6-phosphate | 259 | 97 | 3.23 | 1.23E-05 | 1.91E-05 | 1.69 | 0.017 | 0.19 | 0.64 | -0.64 |  |
| 34 | D-glutamine | 147.1 | 84 | 0.66 | 1.60E-02 | 1.16E-02 | 1.74 | 0.009 | 0.15 | 1.38 | 0.47 |  |
| 35 | D-maltose | 341.1 | 59 | 0.76 | 2.25E-02 | 3.26E-02 | 1.65 | 0.019 | 0.20 | 0.69 | -0.54 |  |
| 36 | D-proline | 116.1 | 70.1 | 0.76 | 1.85E-02 | 3.20E-02 | 1.87 | 0.000 | 0.000 | 0.58 | -0.79 |  |
| 37 | Deethylatrazine | 188.1 | 146 | 3.81 | 1.35E-02 | 7.96E-03 | 1.74 | 0.007 | 0.14 | 1.70 | 0.76 |  |
| 38 | Delphinidin-3-O-glucoside | 465.1 | 303.1 | 6.06 | 2.00E-05 | 3.26E-05 | 1.69 | 0.012 | 0.17 | 0.61 | -0.71 |  |
| 39 | Deltonin;gracillin | 885.5 | 723.4 | 7.13 | 1.62E-05 | 4.70E-05 | 1.83 | 0.003 | 0.10 | 0.34 | -1.54 |  |
| 40 | Diacetoxy-6-gingerdiol | 379.2 | 59 | 4.51 | 4.60E-05 | 6.83E-05 | 1.64 | 0.022 | 0.21 | 0.67 | -0.57 |  |
| 41 | Diallyl disulfide | 147 | 41 | 0.68 | 9.19E-04 | 6.22E-04 | 1.78 | 0.032 | 0.24 | 1.48 | 0.56 |  |
| 42 | Diphyllin | 381.1 | 335.1 | 10.09 | 5.08E-06 | 2.09E-06 | 1.61 | 0.046 | 0.27 | 2.43 | 1.28 |  |
| 43 | DL-alpha-Tocopherylacetate | 473.4 | 207.1 | 13.37 | 4.30E-06 | 7.03E-06 | 1.81 | 0.001 | 0.058 | 0.61 | -0.71 |  |
| 44 | Ethyl acrylate | 101.1 | 55 | 1.74 | 1.18E-04 | 1.82E-04 | 1.72 | 0.014 | 0.18 | 0.65 | -0.63 |  |
| 45 | Ethyl caproate | 145.1 | 57.1 | 0.74 | 2.27E-04 | 4.16E-04 | 1.75 | 0.004 | 0.11 | 0.55 | -0.87 |  |
| 46 | Exemestane | 297.2 | 121.1 | 11.61 | 2.62E-04 | 1.72E-04 | 1.67 | 0.029 | 0.23 | 1.53 | 0.61 |  |
| 47 | Fingolimod hydrochloride | 308.3 | 105.1 | 13.11 | 3.56E-05 | 6.49E-05 | 1.43 | 0.042 | 0.26 | 0.55 | -0.87 |  |
| 48 | Ganoderic acid S | 453.3 | 391.3 | 9.21 | 1.96E-05 | 1.27E-04 | 1.83 | 0.001 | 0.07 | 0.15 | -2.70 |  |
| 49 | Genipin-1-O-gentiobioside | 551.2 | 515.2 | 4.67 | 4.69E-06 | 8.99E-06 | 1.43 | 0.027 | 0.23 | 0.52 | -0.94 |  |
| 50 | Homopterocarpin | 285.1 | 107 | 9.07 | 7.40E-05 | 2.79E-05 | 1.51 | 0.007 | 0.14 | 2.65 | 1.41 |  |
| 51 | Hordenine | 166.1 | 121.1 | 3.04 | 1.47E-03 | 1.07E-03 | 1.58 | 0.032 | 0.24 | 1.37 | 0.46 |  |
| 52 | Indole | 118.1 | 91.1 | 3.82 | 1.13E-02 | 8.78E-03 | 1.60 | 0.040 | 0.26 | 1.28 | 0.36 |  |
| 53 | Isoquercitrin | 465.4 | 302.8 | 6.06 | 2.00E-05 | 3.26E-05 | 1.69 | 0.012 | 0.17 | 0.61 | -0.71 |  |
| 54 | Isoscoparin | 463.1 | 445.1 | 6.23 | 1.15E-05 | 2.01E-05 | 1.65 | 0.020 | 0.20 | 0.57 | -0.81 |  |
| 55 | Jasmonic acid | 211.1 | 151.1 | 6.73 | 6.21E-05 | 4.57E-05 | 1.75 | 0.045 | 0.27 | 1.36 | 0.44 |  |
| 56 | Kaempferol 3-O-beta-sophoroside | 611.2 | 287.1 | 6.83 | 2.29E-04 | 3.31E-04 | 1.70 | 0.012 | 0.17 | 0.69 | -0.53 |  |
| 57 | L-asparagine | 133.1 | 74 | 0.64 | 6.88E-04 | 4.31E-04 | 1.59 | 0.046 | 0.27 | 1.59 | 0.67 |  |
| 58 | L-homocitrulline | 190.1 | 101.1 | 0.72 | 5.38E-05 | 2.66E-05 | 1.75 | 0.011 | 0.16 | 2.02 | 1.02 |  |
| 59 | L-lysine;l-glutamine | 147.1 | 84.067 | 0.64 | 1.60E-02 | 1.16E-02 | 1.74 | 0.009 | 0.15 | 1.38 | 0.47 |  |
| 60 | L-phenylalanine | 166.1 | 120.1 | 2.82 | 2.30E-02 | 8.13E-03 | 1.17 | 0.022 | 0.21 | 2.83 | 1.50 |  |
| 61 | L-Pipecolic acid | 130.1 | 84.1 | 0.69 | 1.44E-02 | 8.88E-03 | 1.85 | 0.000 | 0.045 | 1.62 | 0.69 |  |
| 62 | L-Pipecolic acid | 130.1 | 84.1 | 1.47 | 3.65E-03 | 2.72E-03 | 1.61 | 0.017 | 0.19 | 1.34 | 0.42 |  |
| 63 | Lansiumarin A | 353.1 | 69 | 11.65 | 1.93E-04 | 2.37E-04 | 1.56 | 0.037 | 0.25 | 0.82 | -0.29 |  |
| 64 | Levamisole | 205.1 | 91.1 | 12.04 | 9.67E-05 | 5.75E-05 | 1.67 | 0.024 | 0.21 | 1.68 | 0.75 |  |
| 65 | Lumichrome | 243.1 | 198.1 | 6.70 | 1.95E-05 | 2.05E-06 | 1.51 | 0.035 | 0.25 | 9.51 | 3.25 |  |
| 66 | Lutein | 569.4 | 533.4 | 13.14 | 4.24E-05 | 2.28E-05 | 1.70 | 0.015 | 0.18 | 1.86 | 0.89 |  |
| 67 | Macamide B | 346.3 | 91.1 | 8.85 | 4.82E-05 | 1.95E-05 | 1.64 | 0.007 | 0.14 | 2.48 | 1.31 |  |
| 68 | Maltotriose | 527.2 | 365.1 | 1.23 | 4.45E-03 | 7.30E-03 | 1.64 | 0.027 | 0.22 | 0.61 | -0.71 |  |
| 69 | Mellein | 179.1 | 161.1 | 6.45 | 6.97E-06 | 4.35E-06 | 1.56 | 0.039 | 0.26 | 1.60 | 0.68 |  |

**Table S3. OPLS-DA model reference table in pepper fruit**

| Type | N | R^2^X | R^2^Y | Q^2^ | Title |
| --- | --- | --- | --- | --- | --- |
| OPLS-DA | 6 | 0.522 | 0.999 | 0.7 | T1 vs CK |
| OPLS-DA | 6 | 0.516 | 1 | 0.825 | T2 vs CK |
| OPLS-DA | 6 | 0.467 | 1 | 0.818 | T3 vs CK |

**Table S4. Differential metabolites were identified in pepper fruit treated with different concentrations of nano-Se**

| Differential metabolites of nano-selenium (1 mg/L) and control in pepper fruits | | | | | | | | | | | |
| --- | --- | --- | --- | --- | --- | --- | --- | --- | --- | --- | --- |
| Number | Compound | Q1 | Q3 | RT | Mean T1 | Mean CK | VIP | P-value | Q-value | Fold Change | LOG_Fold Change |
| 1 | (-)-Anonaine | 266.1 | 192.1 | 7.4233 | 4.09E-05 | 1.18E-04 | 1.76 | 0.01 | 0.46 | 0.35 | -1.53 |
| 2 | 1-(alpha-Methyl-4-(2-methylpropyl)benzeneacetate)-beta-D-Glucopyranuronic acid | 383.2 | 189.1 | 12.18463 | 4.45E-04 | 2.04E-04 | 1.76 | 0.02 | 0.47 | 2.18 | 1.12 |
| 3 | 1-phenylethanol | 123.1 | 105.1 | 10.55668 | 1.70E-04 | 3.34E-04 | 1.65 | 0.05 | 0.50 | 0.51 | -0.98 |
| 4 | 22-dehydroclerosterol | 411.4 | 93.1 | 12.58742 | 5.30E-03 | 3.11E-03 | 1.55 | 0.05 | 0.50 | 1.70 | 0.77 |
| 5 | 3-Carbamyl-1-methylpyridinium (1-Methylnicotinamide) | 137.1 | 94.1 | 6.359117 | 4.74E-03 | 2.39E-03 | 1.69 | 0.05 | 0.50 | 1.98 | 0.99 |
| 6 | 3,9-dihydroxypterocarpan | 257.1 | 95 | 9.650417 | 2.79E-04 | 1.23E-04 | 1.73 | 0.01 | 0.46 | 2.28 | 1.19 |
| 7 | 4-Aminobutyric acid | 104.1 | 87.1 | 0.66 | 3.37E-03 | 1.30E-03 | 1.73 | 0.03 | 0.49 | 2.60 | 1.38 |
| 8 | 4-Hydroxyphenylacetylglutamic acid | 282.1 | 107 | 12.9902 | 2.91E-01 | 1.99E-01 | 1.82 | 0.00 | 0.42 | 1.46 | 0.55 |
| 9 | Acetylcholine chloride | 182.1 | 122.1 | 3.306483 | 4.36E-05 | 9.98E-05 | 1.57 | 0.03 | 0.48 | 0.44 | -1.20 |
| 10 | Alantolactone | 233.2 | 105.1 | 11.93288 | 9.10E-04 | 5.53E-04 | 1.71 | 0.02 | 0.47 | 1.65 | 0.72 |
| 11 | Anisatin | 329.1 | 311.1 | 5.422883 | 6.47E-04 | 2.96E-04 | 1.68 | 0.03 | 0.49 | 2.19 | 1.13 |
| 12 | Anthranilic acid | 138.1 | 120 | 2.87835 | 8.89E-04 | 4.62E-04 | 1.87 | 0.00 | 0.42 | 1.92 | 0.94 |
| 13 | Anthraquinone | 209.1 | 105 | 8.075917 | 1.70E-04 | 3.28E-04 | 1.78 | 0.02 | 0.47 | 0.52 | -0.95 |
| 14 | Apiin | 565.5 | 432.9 | 6.46 | 1.20E-02 | 1.12E-03 | 1.54 | 0.02 | 0.47 | 10.78 | 3.43 |
| 15 | Aristolindiquinone | 219.1 | 105 | 11.14408 | 1.28E-03 | 4.68E-04 | 1.79 | 0.00 | 0.42 | 2.75 | 1.46 |
| 16 | Baicalein | 271.1 | 123 | 9.38 | 2.94E-03 | 1.58E-03 | 1.68 | 0.04 | 0.49 | 1.86 | 0.89 |
| 17 | Chamazulene | 185.1 | 157.1 | 8.741517 | 2.82E-04 | 5.36E-04 | 1.56 | 0.02 | 0.47 | 0.53 | -0.93 |
| 18 | Chelidonic acid | 185 | 69 | 1.388967 | 3.23E-03 | 2.22E-03 | 1.53 | 0.05 | 0.50 | 1.46 | 0.55 |
| 19 | D-arabinose | 149 | 59 | 0.747958 | 1.20E-03 | 6.88E-04 | 1.63 | 0.02 | 0.47 | 1.74 | 0.80 |
| 20 | D-Aspartic acid | 134 | 74 | 0.755483 | 1.09E-02 | 6.89E-03 | 1.77 | 0.02 | 0.47 | 1.58 | 0.66 |
| 21 | D-glutamine | 147.1 | 84 | 0.658483 | 5.14E-01 | 3.29E-01 | 1.71 | 0.04 | 0.49 | 1.56 | 0.64 |
| 22 | D-ribose | 149 | 59 | 0.7736 | 1.20E-03 | 6.88E-04 | 1.63 | 0.02 | 0.47 | 1.74 | 0.80 |
| 23 | Deguelin | 395.1 | 147 | 13.46013 | 5.50E-04 | 3.39E-04 | 1.66 | 0.02 | 0.47 | 1.62 | 0.70 |
| 24 | Diallyl disulfide | 147 | 41 | 0.678083 | 3.67E-02 | 2.34E-02 | 1.70 | 0.04 | 0.50 | 1.56 | 0.65 |
| 25 | Dihydrocapsaicin | 308.2 | 137.1 | 11.93663 | 2.29E+00 | 1.72E+00 | 1.60 | 0.04 | 0.49 | 1.33 | 0.42 |
| 26 | Dimethylbenzimidazole | 147.1 | 131.1 | 10.5638 | 1.84E-04 | 3.56E-04 | 1.72 | 0.02 | 0.47 | 0.52 | -0.95 |
| 27 | Diosmetin | 301.1 | 258.1 | 9.311889 | 2.05E-03 | 9.35E-04 | 1.68 | 0.04 | 0.50 | 2.19 | 1.13 |
| 28 | L-alanine | 90.1 | 44 | 0.644928 | 3.72E-03 | 2.21E-03 | 1.82 | 0.01 | 0.45 | 1.68 | 0.75 |
| 29 | Enol-phenylpyruvate | 165.1 | 91.1 | 5.0757 | 1.09E-03 | 6.91E-04 | 1.92 | 0.00 | 0.42 | 1.57 | 0.65 |
| 30 | Furan-3-carboxylic acid | 111 | 67 | 13.77197 | 4.96E-05 | 2.71E-05 | 1.83 | 0.01 | 0.42 | 1.83 | 0.87 |
| 31 | Guggulsterone E&Z | 343.2 | 325.2 | 11.07695 | 4.81E-05 | 1.33E-04 | 1.48 | 0.03 | 0.49 | 0.36 | -1.46 |
| 32 | Hydrocinnamic acid | 151.1 | 45 | 11.247 | 1.50E-04 | 8.86E-05 | 1.62 | 0.03 | 0.48 | 1.70 | 0.76 |
| 33 | Indole | 118.1 | 91.1 | 3.81955 | 1.45E-01 | 1.04E-01 | 1.63 | 0.04 | 0.50 | 1.39 | 0.47 |
| 34 | L-Aspartic acid | 134 | 74 | 0.63 | 1.09E-02 | 6.89E-03 | 1.77 | 0.02 | 0.47 | 1.58 | 0.66 |
| 35 | Morusin | 421.2 | 403.2 | 4.90085 | 6.86E-04 | 3.96E-04 | 1.68 | 0.05 | 0.51 | 1.73 | 0.79 |
| 36 | Myrcene | 137.1 | 53 | 11.73747 | 2.66E-02 | 1.91E-02 | 1.67 | 0.04 | 0.49 | 1.39 | 0.48 |
| 37 | Myristoleic acid | 227.2 | 69.1 | 4.959967 | 9.93E-04 | 4.53E-04 | 1.76 | 0.01 | 0.45 | 2.19 | 1.13 |
| 38 | Naringenin chalcone | 273.1 | 153 | 6.54065 | 2.21E-03 | 7.75E-04 | 1.67 | 0.02 | 0.46 | 2.85 | 1.51 |
| 39 | O-acetylethanolamine | 104.1 | 44 | 0.671567 | 1.20E-01 | 8.12E-02 | 1.68 | 0.04 | 0.49 | 1.48 | 0.57 |
| 40 | O-phosphorylethanolamine | 140 | 79 | 0.69215 | 5.69E-03 | 1.22E-03 | 1.43 | 0.04 | 0.49 | 4.67 | 2.22 |
| 41 | Phosphoric acid | 99 | 81 | 0.64835 | 7.71E-02 | 6.30E-02 | 1.83 | 0.01 | 0.44 | 1.22 | 0.29 |
| 42 | Psoralidin | 337.1 | 55.1 | 11.79862 | 8.16E-04 | 1.15E-03 | 1.81 | 0.00 | 0.42 | 0.71 | -0.50 |
| 43 | Scoparone | 207.1 | 121 | 5.059167 | 1.74E-03 | 7.32E-04 | 1.69 | 0.04 | 0.50 | 2.38 | 1.25 |
| 44 | Withanolide A | 471.3 | 453.3 | 12.26855 | 1.96E-04 | 4.55E-04 | 1.46 | 0.05 | 0.50 | 0.43 | -1.21 |

| Differential metabolites of nano-selenium (5 mg/L) and control in pepper fruits | | | | | | | | | | | | | |
| --- | --- | --- | --- | --- | --- | --- | --- | --- | --- | --- | --- | --- | --- |
| Number | Compound | | Q1 | Q3 | RT | Mean T1 | Mean CK | VIP | P-value | Q-value | Fold Change | LOG_Fold Change |  |
| 1 | (S)-2-Aceto-2-hydroxybutanoate | 147.1 | | 57 | 0.66 | 5.20E-03 | 2.89E-03 | 1.64 | 0.01 | 0.11 | 1.80 | 0.85 |  |
| 2 | 2-hydroxyethanesulfonate | 127 | | 109 | 0.76 | 1.13E-03 | 7.39E-04 | 1.55 | 0.02 | 0.14 | 1.52 | 0.61 |  |
| 3 | 3-dehydroquinate | 191.1 | | 145.1 | 10.56 | 4.21E-04 | 2.32E-04 | 1.65 | 0.01 | 0.09 | 1.82 | 0.86 |  |
| 4 | 3-Hydroxy-4-methoxycinnamic acid;Isoferulic acid | 195.1 | | 149.1 | 4.87 | 2.80E-03 | 1.96E-03 | 1.56 | 0.02 | 0.13 | 1.43 | 0.52 |  |
| 5 | 3-Octyl alcohol | 131.1 | | 113.1 | 1.23 | 9.13E-04 | 6.52E-04 | 1.52 | 0.03 | 0.16 | 1.40 | 0.49 |  |
| 6 | 4-Aminobutyric acid | 104.1 | | 87.1 | 0.66 | 2.40E-03 | 1.30E-03 | 1.54 | 0.02 | 0.14 | 1.85 | 0.89 |  |
| 7 | 4-sulfobenzoate | 203 | | 157 | 4.68 | 1.56E-03 | 8.36E-04 | 1.52 | 0.04 | 0.18 | 1.86 | 0.90 |  |
| 8 | 5-aminolevulinate | 132.1 | | 55 | 1.56 | 4.62E-04 | 2.88E-04 | 1.76 | 0.00 | 0.02 | 1.60 | 0.68 |  |
| 9 | 5-Aminovaleric acid | 118.1 | | 55.1 | 1.11 | 1.70E-02 | 9.97E-03 | 1.70 | 0.01 | 0.09 | 1.71 | 0.77 |  |
| 10 | 6-Amino-2-oxohexanoate | 146.1 | | 72.1 | 0.77 | 2.80E-01 | 2.05E-01 | 1.78 | 0.01 | 0.09 | 1.37 | 0.45 |  |
| 11 | 6-Aminocaproic acid | 132.1 | | 41 | 1.84 | 4.22E-03 | 2.50E-03 | 1.63 | 0.02 | 0.14 | 1.69 | 0.76 |  |
| 12 | 7-methylguanine | 166.1 | | 149 | 1.56 | 6.83E-04 | 2.98E-04 | 1.41 | 0.05 | 0.20 | 2.29 | 1.20 |  |
| 13 | Allysine(6-oxo dl-norleucine) | 146.1 | | 100.1 | 0.77 | 2.10E+00 | 1.47E+00 | 1.72 | 0.01 | 0.08 | 1.43 | 0.52 |  |
| 14 | Alpha-d-glucose | 163.1 | | 85 | 0.76 | 1.74E-03 | 9.10E-04 | 1.52 | 0.03 | 0.15 | 1.92 | 0.94 |  |
| 15 | Apiin | 565.5 | | 432.9 | 6.46 | 5.52E-03 | 1.12E-03 | 1.35 | 0.02 | 0.14 | 4.94 | 2.31 |  |
| 16 | Aristolindiquinone | 219.1 | | 105 | 11.14 | 8.38E-04 | 4.68E-04 | 1.47 | 0.03 | 0.16 | 1.79 | 0.84 |  |
| 17 | Ascorbic acid | 177 | | 95 | 1.13 | 3.39E-02 | 2.81E-02 | 1.59 | 0.02 | 0.14 | 1.21 | 0.27 |  |
| 18 | Bergamotine | 339.2 | | 203 | 13.03 | 5.10E-04 | 1.42E-04 | 1.66 | 0.02 | 0.14 | 3.60 | 1.85 |  |
| 19 | Bovinic acid | 303.2 | | 183 | 6.86 | 9.03E-05 | 2.88E-04 | 1.60 | 0.03 | 0.16 | 0.31 | -1.68 |  |
| 20 | Butein | 273.1 | | 137 | 7.07 | 1.35E-04 | 8.35E-05 | 1.51 | 0.04 | 0.17 | 1.62 | 0.70 |  |
| 21 | Cis-4-Hydroxy-D-proline | 130.1 | | 45 | 0.65 | 1.69E-05 | 2.33E-04 | 1.65 | 0.01 | 0.09 | 0.07 | -3.79 |  |
| 22 | Cis-Aconitic acid | 175 | | 69 | 1.46 | 4.11E-02 | 2.45E-02 | 1.60 | 0.03 | 0.15 | 1.68 | 0.75 |  |
| 23 | Citric acid | 191 | | 111 | 3.65 | 5.03E-04 | 2.89E-04 | 1.43 | 0.05 | 0.20 | 1.75 | 0.80 |  |
| 24 | Cupressuflavone | 539.1 | | 521.1 | 9.67 | 1.32E-04 | 2.92E-04 | 1.68 | 0.01 | 0.08 | 0.45 | -1.15 |  |
| 25 | Cyanidin-3-O-rhamnoside chloride | 434.1 | | 271.1 | 7.32 | 4.26E-04 | 1.21E-04 | 1.57 | 0.04 | 0.17 | 3.52 | 1.81 |  |
| 26 | Cysteinylglycine | 179 | | 76 | 1.38 | 1.83E-02 | 1.38E-02 | 1.59 | 0.02 | 0.12 | 1.32 | 0.40 |  |
| 27 | D-alpha-Aminobutyric acid | 104.1 | | 58.1 | 0.65 | 3.35E-01 | 2.42E-01 | 1.67 | 0.01 | 0.09 | 1.38 | 0.47 |  |
| 28 | D-Aspartic acid | 134 | | 74 | 0.76 | 1.63E-02 | 6.89E-03 | 1.78 | 0.00 | 0.02 | 2.37 | 1.25 |  |
| 29 | D-glutamine | 147.1 | | 84 | 0.66 | 7.16E-01 | 3.29E-01 | 1.78 | 0.00 | 0.02 | 2.18 | 1.12 |  |
| 30 | D-proline | 116.1 | | 70.1 | 0.76 | 2.86E+00 | 3.19E+00 | 1.56 | 0.03 | 0.15 | 0.90 | -0.16 |  |
| 31 | D-serine | 106 | | 60 | 0.66 | 9.94E-03 | 6.03E-03 | 1.73 | 0.01 | 0.08 | 1.65 | 0.72 |  |
| 32 | Deethylatrazine | 188.1 | | 146 | 3.81 | 8.22E-02 | 4.51E-02 | 1.79 | 0.00 | 0.02 | 1.82 | 0.87 |  |
| 33 | Dexmedetomidine | 201.1 | | 105.1 | 10.51 | 1.02E-04 | 4.22E-04 | 1.74 | 0.00 | 0.05 | 0.24 | -2.04 |  |
| 34 | Diallyl disulfide | 147 | | 41 | 0.68 | 5.29E-02 | 2.34E-02 | 1.78 | 0.00 | 0.02 | 2.25 | 1.17 |  |
| 35 | L-alanine | 90.1 | | 44 | 0.64 | 3.69E-03 | 2.21E-03 | 1.74 | 0.00 | 0.07 | 1.67 | 0.74 |  |
| 36 | Gluconic acid | 195.1 | | 75 | 0.69 | 1.53E-02 | 7.97E-03 | 1.58 | 0.03 | 0.16 | 1.92 | 0.94 |  |
| 37 | Homobaldrinal | 261.1 | | 159 | 10.00 | 2.03E-04 | 1.14E-04 | 1.53 | 0.03 | 0.15 | 1.78 | 0.83 |  |
| 38 | Homoorientin | 449 | | 430.9 | 5.43 | 4.73E-03 | 8.43E-03 | 1.73 | 0.01 | 0.07 | 0.56 | -0.83 |  |
| 39 | Indole | 118.1 | | 91.1 | 3.82 | 1.57E-01 | 1.04E-01 | 1.66 | 0.01 | 0.08 | 1.51 | 0.59 |  |
| 40 | Isofraxidin | 223.1 | | 205.1 | 4.25 | 6.46E-04 | 3.67E-04 | 1.56 | 0.03 | 0.16 | 1.76 | 0.81 |  |
| 41 | Isoleucine | 132.1 | | 86.1 | 1.80 | 1.82E-03 | 1.41E-03 | 1.70 | 0.01 | 0.08 | 1.29 | 0.36 |  |
| 42 | Isosakuranetin | 287.1 | | 153 | 6.53 | 3.80E-03 | 7.21E-03 | 1.60 | 0.03 | 0.16 | 0.53 | -0.93 |  |
| 43 | Kaurenoic acid | 303.2 | | 257.2 | 5.90 | 9.11E-05 | 6.11E-04 | 1.58 | 0.02 | 0.12 | 0.15 | -2.75 |  |
| 44 | L-arginine | 175.1 | | 70.05 | 0.6 | 2.85E+00 | 1.62E+00 | 1.67 | 0.01 | 0.10 | 1.76 | 0.82 |  |
| 45 | L-asparagine | 133.1 | | 74 | 0.64 | 1.07E-01 | 7.07E-02 | 1.71 | 0.01 | 0.08 | 1.52 | 0.60 |  |
| 46 | L-Aspartic acid | 134 | | 74 | 0.63 | 1.54E-02 | 6.89E-03 | 1.79 | 0.00 | 0.02 | 2.23 | 1.16 |  |
| 47 | L-citruline | 176.1 | | 70.1 | 0.66 | 1.20E-01 | 7.98E-02 | 1.52 | 0.03 | 0.15 | 1.50 | 0.59 |  |
| 48 | L-Glutamic acid | 148.1 | | 84 | 0.66 | 1.28E-01 | 6.73E-02 | 1.78 | 0.01 | 0.10 | 1.90 | 0.92 |  |
| 49 | L-glutamine | 145.1 | | 42 | 0.64 | 5.03E-02 | 2.51E-02 | 1.72 | 0.00 | 0.03 | 2.00 | 1.00 |  |
| 50 | L-histidine | 156.1 | | 110.1 | 0.59 | 5.74E-02 | 4.01E-02 | 1.72 | 0.03 | 0.16 | 1.43 | 0.52 |  |
| 51 | L-homoserine | 120.1 | | 56 | 0.67 | 1.94E-02 | 1.25E-02 | 1.79 | 0.00 | 0.02 | 1.55 | 0.63 |  |
| 52 | L-glutamine | 147.1 | | 84.1 | 0.64 | 8.50E-01 | 3.97E-01 | 1.77 | 0.02 | 0.13 | 2.14 | 1.10 |  |
| 53 | L-methionine | 150.05 | | 56.1 | 0.94 | 5.40E-03 | 2.47E-03 | 1.70 | 0.01 | 0.08 | 2.19 | 1.13 |  |
| 54 | L-norleucine | 132.1 | | 86.1 | 1.81 | 1.82E-03 | 1.41E-03 | 1.70 | 0.01 | 0.08 | 1.29 | 0.36 |  |
| 55 | L-phenylalanine | 166.1 | | 120.1 | 2.82 | 3.29E-01 | 2.33E-01 | 1.70 | 0.01 | 0.09 | 1.41 | 0.50 |  |
| 56 | L-Pipecolic acid | 130.1 | | 84.1 | 0.69 | 6.35E-01 | 3.32E-01 | 1.79 | 0.00 | 0.02 | 1.91 | 0.93 |  |
| 57 | L-serine | 106 | | 60 | 0.62 | 9.94E-03 | 6.03E-03 | 1.73 | 0.01 | 0.08 | 1.65 | 0.72 |  |
| 58 | L-theanine | 175.1 | | 84 | 0.85 | 3.01E-03 | 1.61E-03 | 1.65 | 0.01 | 0.08 | 1.87 | 0.90 |  |
| 59 | L-threonine | 120.1 | | 56.1 | 0.64 | 1.94E-02 | 1.25E-02 | 1.79 | 0.00 | 0.02 | 1.55 | 0.63 |  |
| 60 | Limonexic acid | 503.2 | | 485.2 | 12.55 | 4.78E-03 | 1.98E-03 | 1.56 | 0.01 | 0.09 | 2.41 | 1.27 |  |
| 61 | Mannose 6-phosphate | 283 | | 185 | 13.03 | 6.27E-03 | 7.72E-03 | 1.64 | 0.02 | 0.12 | 0.81 | -0.30 |  |
| 62 | Muramic acid | 252.1 | | 72 | 2.78 | 3.11E-03 | 2.15E-03 | 1.52 | 0.05 | 0.20 | 1.44 | 0.53 |  |
| 63 | Myrcene | 137.1 | | 53 | 11.74 | 2.31E-02 | 1.91E-02 | 1.57 | 0.02 | 0.14 | 1.21 | 0.28 |  |
| 64 | N6-Acetyl-L-lysine | 189.1 | | 84.1 | 0.81 | 6.95E-01 | 5.30E-01 | 1.62 | 0.01 | 0.11 | 1.31 | 0.39 |  |
| 65 | Nobiletin | 403.1 | | 373.1 | 11.03 | 6.32E-03 | 5.02E-03 | 1.68 | 0.01 | 0.10 | 1.26 | 0.33 |  |
| 66 | O-acetylethanolamine | 104.1 | | 44 | 0.67 | 1.16E-01 | 8.12E-02 | 1.58 | 0.03 | 0.15 | 1.43 | 0.52 |  |
| 67 | Phenylpyruvic acid | 163 | | 91.1 | 5.67 | 3.61E-04 | 1.73E-04 | 1.62 | 0.01 | 0.11 | 2.09 | 1.07 |  |
| 68 | Phloretic acid | 167.1 | | 121.1 | 2.84 | 4.53E-02 | 2.81E-02 | 1.58 | 0.03 | 0.16 | 1.61 | 0.69 |  |
| 69 | Phosphoric acid | 99 | | 81 | 0.65 | 5.33E-02 | 6.30E-02 | 1.75 | 0.00 | 0.04 | 0.85 | -0.24 |  |
| 70 | L-proline | 116.1 | | 70.1 | 0.73 | 2.86E+00 | 3.19E+00 | 1.56 | 0.03 | 0.15 | 0.90 | -0.16 |  |
| 71 | Pseudotropine | 142.1 | | 124.1 | 0.81 | 1.05E-02 | 1.32E-02 | 1.58 | 0.02 | 0.13 | 0.79 | -0.33 |  |
| 72 | Psoralidin | 337.1 | | 55.1 | 11.80 | 7.25E-04 | 1.15E-03 | 1.77 | 0.00 | 0.02 | 0.63 | -0.67 |  |
| 73 | Pyrrolidonecarboxylic acid | 130 | | 84 | 0.67 | 6.35E-01 | 3.32E-01 | 1.79 | 0.00 | 0.02 | 1.91 | 0.93 |  |
| 74 | Rutin | 611 | | 465 | 5.85 | 6.15E-04 | 4.44E-03 | 1.59 | 0.00 | 0.06 | 0.14 | -2.85 |  |
| 75 | S-lactoylglutathione | 380.1 | | 262.1 | 4.09 | 7.21E-05 | 2.59E-05 | 1.46 | 0.03 | 0.16 | 2.79 | 1.48 |  |
| 76 | Trans-4-Hydroxy-L-proline | 132.1 | | 86.05 | 0.65 | 4.50E-03 | 2.49E-03 | 1.65 | 0.01 | 0.09 | 1.81 | 0.85 |  |
| 77 | L-tryptophan | 205.1 | | 188.1 | 3.77 | 2.54E-03 | 1.37E-03 | 1.65 | 0.02 | 0.13 | 1.85 | 0.89 |  |
| 78 | Vasicine | 189.1 | | 171.1 | 3.76 | 4.01E-03 | 2.14E-03 | 1.69 | 0.01 | 0.08 | 1.88 | 0.91 |  |

| Differential metabolites of nano-selenium (20 mg/L) and control in pepper fruits | | | | | | | | | | | |
| --- | --- | --- | --- | --- | --- | --- | --- | --- | --- | --- | --- |
| Number | Compound | Q1 | Q3 | RT | Mean T1 | Mean CK | VIP | P-value | Q-value | Fold Change | LOG_Fold Change |
| 1 | (10E,12Z)-(9S)-9-Hydroperoxyoctadeca-10,12-dienoic acid | 295.2 | 55.1 | 11.96 | 1.00E-03 | 4.18E-04 | 1.57 | 0.034 | 0.38 | 2.39 | 1.26 |
| 2 | (S)-2-Aceto-2-hydroxybutanoate | 147.1 | 57 | 0.66 | 4.78E-03 | 2.89E-03 | 1.76 | 0.006 | 0.17 | 1.65 | 0.73 |
| 3 | 13-L-Hydroperoxylinoleic acid | 295.2 | 55.1 | 11.98 | 1.00E-03 | 4.18E-04 | 1.57 | 0.034 | 0.38 | 2.39 | 1.26 |
| 4 | 3,4-Dihydroxyphenylacetaldehyde | 153.1 | 135 | 4.05 | 2.25E-04 | 1.51E-04 | 1.64 | 0.027 | 0.35 | 1.49 | 0.57 |
| 5 | 4-Hydroxyphenylacetic acid | 151 | 107 | 9.38 | 1.06E-04 | 7.46E-05 | 1.63 | 0.033 | 0.37 | 1.42 | 0.50 |
| 6 | 4-Hydroxyphenylacetylglutamic acid | 282.1 | 107 | 12.99 | 2.83E-01 | 1.99E-01 | 1.65 | 0.032 | 0.37 | 1.43 | 0.51 |
| 7 | 4'-Demethylpodophyllotoxin | 401.1 | 383.1 | 6.43 | 1.57E-04 | 4.11E-04 | 1.76 | 0.029 | 0.36 | 0.38 | -1.39 |
| 8 | 6-Amino-2-oxohexanoate | 146.1 | 72.1 | 0.77 | 2.74E-01 | 2.05E-01 | 1.84 | 0.028 | 0.36 | 1.34 | 0.42 |
| 9 | 6-Aminocaproic acid | 132.1 | 41 | 1.84 | 3.92E-03 | 2.50E-03 | 1.61 | 0.048 | 0.40 | 1.57 | 0.65 |
| 10 | alpha-D-Glucose | 163.1 | 85 | 0.76 | 2.26E-03 | 9.10E-04 | 1.72 | 0.015 | 0.29 | 2.48 | 1.31 |
| 11 | Amabiline | 284.2 | 122.1 | 12.99 | 2.48E-03 | 1.87E-03 | 1.58 | 0.044 | 0.40 | 1.32 | 0.41 |
| 12 | Arachidonic acid | 305.2 | 93.1 | 12.40 | 3.93E-04 | 1.13E-04 | 1.72 | 0.042 | 0.39 | 3.49 | 1.80 |
| 13 | Behenic acid | 339.3 | 183 | 13.09 | 3.43E-01 | 4.98E-01 | 1.68 | 0.028 | 0.36 | 0.69 | -0.54 |
| 14 | beta-Nicotinamide mononucleotide | 335.1 | 123.1 | 0.73 | 2.06E-03 | 1.05E-03 | 1.73 | 0.009 | 0.22 | 1.96 | 0.97 |
| 15 | Bovinic acid | 303.2 | 183 | 6.86 | 1.07E-04 | 2.88E-04 | 1.64 | 0.049 | 0.41 | 0.37 | -1.43 |
| 16 | Chalconaringenin | 272.9 | 152.9 | 8.78 | 1.63E-01 | 2.52E-01 | 1.85 | 0.001 | 0.09 | 0.65 | -0.62 |
| 17 | Cinchonine | 295.2 | 79.1 | 11.99 | 1.73E-03 | 6.82E-04 | 1.68 | 0.031 | 0.36 | 2.53 | 1.34 |
| 18 | Cyanidin 3-rutinoside | 595.2 | 287.1 | 6.08 | 3.62E-03 | 1.67E-02 | 1.44 | 0.028 | 0.36 | 0.22 | -2.21 |
| 19 | Cytidine | 244.1 | 112.1 | 0.70 | 6.67E-04 | 2.76E-04 | 1.55 | 0.040 | 0.39 | 2.42 | 1.27 |
| 20 | D-Aspartic acid | 134 | 74 | 0.76 | 1.02E-02 | 6.89E-03 | 1.69 | 0.027 | 0.35 | 1.47 | 0.56 |
| 21 | D-Glucose 6-phosphate | 259 | 97 | 0.65 | 3.09E-02 | 2.66E-02 | 1.64 | 0.032 | 0.37 | 1.16 | 0.22 |
| 22 | D-Glutamine | 147.1 | 84 | 0.66 | 7.19E-01 | 3.29E-01 | 1.86 | 0.004 | 0.13 | 2.18 | 1.13 |
| 23 | D-Proline | 116.1 | 70.1 | 0.76 | 1.48E+00 | 3.19E+00 | 1.89 | 0.000 | 0.03 | 0.46 | -1.11 |
| 24 | D-Serine | 106 | 60 | 0.66 | 8.55E-03 | 6.03E-03 | 1.64 | 0.044 | 0.40 | 1.42 | 0.50 |
| 25 | Dexmedetomidine | 201.1 | 105.1 | 10.51 | 9.86E-05 | 4.22E-04 | 1.83 | 0.003 | 0.11 | 0.23 | -2.10 |
| 26 | Diallyl disulfide | 147 | 41 | 0.68 | 5.25E-02 | 2.34E-02 | 1.91 | 0.000 | 0.01 | 2.24 | 1.16 |
| 27 | L-Alanine | 90.1 | 44 | 0.64 | 1.53E-03 | 2.21E-03 | 1.79 | 0.006 | 0.16 | 0.69 | -0.53 |
| 28 | Exemestane | 297.2 | 121.1 | 11.61 | 2.60E-04 | 5.60E-04 | 1.73 | 0.031 | 0.37 | 0.46 | -1.11 |
| 29 | Ferulic acid | 195.1 | 117 | 4.86 | 9.74E-03 | 6.05E-03 | 1.70 | 0.030 | 0.36 | 1.61 | 0.69 |
| 30 | Gentioflavin | 194.1 | 148.1 | 4.93 | 1.27E-04 | 3.11E-04 | 1.57 | 0.036 | 0.38 | 0.41 | -1.30 |
| 31 | Homoorientin | 449 | 430.9 | 5.43 | 1.30E-02 | 8.43E-03 | 1.71 | 0.022 | 0.33 | 1.55 | 0.63 |
| 32 | Itaconic acid | 131 | 85 | 0.52 | 4.91E-02 | 2.33E-02 | 1.91 | 0.000 | 0.01 | 2.11 | 1.08 |
| 33 | L-Asparagine | 133.1 | 74 | 0.64 | 7.94E-02 | 7.07E-02 | 1.62 | 0.036 | 0.38 | 1.12 | 0.17 |
| 34 | L-Aspartic acid | 134 | 74 | 0.63 | 1.02E-02 | 6.89E-03 | 1.69 | 0.027 | 0.35 | 1.47 | 0.56 |
| 35 | L-Citruline | 176.1 | 70.1 | 0.66 | 1.26E-01 | 7.98E-02 | 1.66 | 0.025 | 0.34 | 1.58 | 0.66 |
| 36 | L-Glutamic acid | 148.1 | 84 | 0.66 | 9.06E-02 | 6.73E-02 | 1.90 | 0.000 | 0.03 | 1.35 | 0.43 |
| 37 | L-Glutamine | 145.1 | 42 | 0.64 | 4.97E-02 | 2.51E-02 | 1.81 | 0.004 | 0.13 | 1.98 | 0.98 |
| 38 | L-Homoserine | 120.1 | 56 | 0.67 | 1.63E-02 | 1.25E-02 | 1.61 | 0.045 | 0.40 | 1.30 | 0.38 |
| 39 | L-Glutamine | 147.1 | 84.1 | 0.64 | 8.43E-01 | 3.97E-01 | 1.88 | 0.018 | 0.31 | 2.13 | 1.09 |
| 40 | L-Serine | 106 | 60 | 0.62 | 8.55E-03 | 6.03E-03 | 1.64 | 0.044 | 0.40 | 1.42 | 0.50 |
| 41 | L-Threonine | 120.1 | 56.1 | 0.64 | 1.63E-02 | 1.25E-02 | 1.61 | 0.045 | 0.40 | 1.30 | 0.38 |
| 42 | Lubiprostone | 391.2 | 149 | 12.75 | 2.05E-01 | 2.98E-01 | 1.76 | 0.011 | 0.24 | 0.69 | -0.54 |
| 43 | Mannose 6-phosphate | 283 | 185 | 13.03 | 5.46E-03 | 7.72E-03 | 1.64 | 0.023 | 0.33 | 0.71 | -0.50 |
| 44 | Methyl linoleate | 295.3 | 55.1 | 12.00 | 1.00E-03 | 4.18E-04 | 1.57 | 0.034 | 0.38 | 2.39 | 1.26 |
| 45 | Muramic acid | 252.1 | 72 | 2.78 | 3.30E-03 | 2.15E-03 | 1.67 | 0.030 | 0.36 | 1.53 | 0.62 |
| 46 | N-Acetyl-L-glutamate 5-semialdehyde | 174.1 | 86.1 | 1.51 | 1.22E-03 | 5.99E-04 | 1.38 | 0.048 | 0.40 | 2.04 | 1.03 |
| 47 | Naringenin | 273.3 | 152.8 | 8.86 | 1.26E-01 | 1.97E-01 | 1.83 | 0.004 | 0.13 | 0.64 | -0.64 |
| 48 | Phosphoric acid | 99 | 81 | 0.65 | 4.89E-02 | 6.30E-02 | 1.82 | 0.003 | 0.12 | 0.78 | -0.37 |
| 49 | L-Proline | 116.1 | 70.1 | 0.73 | 1.48E+00 | 3.19E+00 | 1.89 | 0.000 | 0.03 | 0.46 | -1.11 |
| 50 | Rutin | 611 | 465 | 5.85 | 7.11E-04 | 4.44E-03 | 1.82 | 0.003 | 0.13 | 0.16 | -2.64 |
| 51 | trans-4-Hydroxy-L-proline;4-Hydroxyproline | 132.1 | 86.05 | 0.65 | 5.49E-03 | 2.49E-03 | 1.83 | 0.001 | 0.09 | 2.20 | 1.14 |
| 52 | Traumatic acid | 229.1 | 81.1 | 8.39 | 1.32E-04 | 2.59E-04 | 1.48 | 0.048 | 0.40 | 0.51 | -0.98 |
| 53 | Uridine | 245.1 | 113 | 2.47 | 1.10E-04 | 2.98E-04 | 1.76 | 0.017 | 0.30 | 0.37 | -1.43 |
| 54 | Uridine 5'-diphospho-D-glucose | 565 | 323 | 0.71 | 1.26E-02 | 8.44E-03 | 1.54 | 0.048 | 0.40 | 1.49 | 0.58 |
| 55 | Withanolide A | 471.3 | 453.3 | 12.27 | 1.30E-04 | 4.55E-04 | 1.78 | 0.002 | 0.10 | 0.29 | -1.81 |

**Table S5. Qualitative identification of compounds in gas phase ion migration spectra**

| Number | Compound | Formula | molecular mass | Relative retention index | RT |  |
| --- | --- | --- | --- | --- | --- | --- |
|  |  |  |  |  |  | Relative migration time |
| 1 | 2-pentenal (E) | C_5_H_8_O | 84.1 | 752.2 | 226.256 | 1.3592 |
| 2 | 2-Hexenal monomer | C_6_H_10_O | 98.1 | 853.5 | 328.561 | 1.18129 |
| 3 | 2-Hexenal dimer | C_6_H_10_O | 98.1 | 854.7 | 329.855 | 1.51264 |
| 4 | hexanal monomer | C_6_H_12_O | 100.2 | 792.8 | 262.059 | 1.26009 |
| 5 | hexanal dimer | C_6_H_12_O | 100.2 | 793.3 | 262.594 | 1.55995 |
| 6 | ethanol monomer | C_2_H_6_O | 46.1 | 512 | 98.849 | 1.04422 |
| 7 | ethanol dimer | C_2_H_6_O | 46.1 | 512.3 | 98.981 | 1.13089 |
| 8 | ethyl acetate | C_4_H_8_O_2_ | 88.1 | 613 | 141.783 | 1.33433 |
| 9 | acetone | C_3_H_6_O | 58.1 | 533.8 | 108.143 | 1.111 |
| 10 | 2,3-butanedione | C_4_H_6_O_2_ | 86.1 | 560 | 119.247 | 1.18145 |
| 11 | Isopropyl alcohol | C_3_H_8_O | 60.1 | 531.5 | 107.163 | 1.21336 |
| 12 | 1-propanol | C_3_H_8_O | 60.1 | 547.7 | 114.021 | 1.24792 |
| 13 | 2-Methyl-2-propenal | C_4_H_6_O | 70.1 | 589.2 | 131.658 | 1.21602 |
| 14 | 2-butanone | C_4_H_8_O | 72.1 | 591.5 | 132.638 | 1.24659 |
| 15 | butanal | C_4_H_8_O | 72.1 | 603 | 137.537 | 1.2878 |
| 16 | Ethyl formate | C_3_H_6_O_2_ | 74.1 | 633 | 150.274 | 1.21602 |
| 17 | 2-methylbutanal | C_5_H_10_O | 86.1 | 671.4 | 166.604 | 1.39681 |
| 18 | 3-methylbutanal | C_5_H_10_O | 86.1 | 654.5 | 159.419 | 1.40479 |
| 19 | 1-penten-3-ol monomer | C_5_H_10_O | 86.1 | 700.3 | 183.523 | 0.94235 |
| 20 | 1-Penten-3-ol dimer | C_5_H_10_O | 86.1 | 697.3 | 181.104 | 1.57362 |
| 21 | 4-heptenal (Z) | C_7_H_12_O | 112.2 | 901.6 | 387.287 | 1.14754 |
| 22 | Isopentyl alcohol | C_5_H_12_O | 88.1 | 738.6 | 215.083 | 1.49838 |
| 23 | 1-pentanol monomer | C_5_H_12_O | 88.1 | 775.4 | 245.39 | 1.25604 |
| 24 | 1-pentanol dimer | C_5_H_12_O | 88.1 | 770.8 | 241.563 | 1.50917 |
| 25 | Ethyl crotonate | C_6_H_10_O_2_ | 114.1 | 840.8 | 314.624 | 1.56371 |
| 26 | 2-heptenal (E) monomer | C_7_H_12_O | 112.2 | 959.9 | 488.556 | 1.26086 |
| 27 | 2-heptenal (E) dimer | C_7_H_12_O | 112.2 | 959.1 | 487.199 | 1.66585 |
| 28 | 1-octen-3-one monomer | C_8_H_14_O | 126.2 | 969.3 | 504.845 | 1.2693 |
| 29 | 1-octen-3-one dimer | C_8_H_14_O | 126.2 | 969 | 504.392 | 1.68554 |
| 30 | 2-Pentylfuran | C_9_H_14_O | 138.2 | 998.8 | 556.876 | 1.25102 |
| 31 | 6-Methyl-5-hepten-2-one | C_8_H_14_O | 126.2 | 997 | 553.256 | 1.17227 |
| 32 | Ethyl hexanoate monomer | C_8_H_16_O_2_ | 144.2 | 1009.7 | 579.046 | 1.34102 |
| 33 | Ethyl hexanoate dimer | C_8_H_16_O_2_ | 144.2 | 1008.1 | 575.879 | 1.80085 |
| 34 | Linalool oxide monomer | C_10_H_18_O_2_ | 170.3 | 1064.1 | 690.088 | 1.25893 |
| 35 | Linalool oxide dimer | C_10_H_18_O_2_ | 170.3 | 1068.1 | 698.318 | 1.81698 |
| 36 | 4-Methyl-1-pentanol | C_6_H_14_O | 102.2 | 840.3 | 314.006 | 1.63264 |
| 37 | (E) -2-nonenal | C_9_H_16_O | 140.2 | 1158.7 | 883.202 | 1.40904 |

**Table S6. Instrumental parameters of three capsaicin compounds in pepper fruit**

| Capsaicinoids | Quantitative ion (m/z) | Qualitative ion (m/z) | RT (min) | CE (V) | Polarity |
| --- | --- | --- | --- | --- | --- |
| Capsaicin | 306.2/137.1 | 306.2/182.1 | 2.64 | 15/5 | Positive |
| Dihydrocapsaicin | 308.2/137 | 308.2/184.2 | 2.79 | 15/5 | Positive |
| Nordihydrocapsaicin | 294.2/136.9 | 294.2/170.1 | 2.59 | 10/5 | Positive |

**Table S7. Analysis conditions of the GC-IMS system**

| GC-IMS unit | |
| --- | --- |
| Analysis time | 30 min |
| Chromatographic column | FS-SE-54-CB-1 15m ID:0.53mm |
| Column temperature | 60℃ |
| Carrier gas/drift gas | N_2_ |
| IMS temperature | 45℃ |
| Automatic headspace injection unit | |
| Injection volume | 500μL |
| Incubation time | 20 min |
| Incubation temperature | 60℃ |
| Injection needle temperature | 85℃ |
| Hatching speed | 500 rpm |
